# Bronze age stone flaking at Saruq al-Hadid, Dubai, southeastern Arabia

**DOI:** 10.1371/journal.pone.0270513

**Published:** 2022-07-13

**Authors:** Mark W. Moore, Lloyd Weeks, Charlotte Cable, Yaaqoub Al-Ali, Mansour Boraik, Hassan Zein

**Affiliations:** 1 Archaeology and Palaeoanthropology, University of New England, Armidale, New South Wales, Australia; 2 Stone Tools and Cognition Research Hub, University of New England, Armidale, New South Wales, Australia; 3 Architectural Heritage and Antiquities Department, Dubai Municipality, United Arab Emirates; Griffith University, AUSTRALIA

## Abstract

Excavations at Saruq al-Hadid, Dubai, UAE, discovered a stone tool technology with backed microliths dating to the Wadi Suq period and Late Bronze Age (ca. 1750–1300 BCE). The stone technology is a contemporary with metal production in the region, and the assemblage was recovered from a thick bone midden deposit at this multi-period site on the edge of the Rub’ al-Khali Desert. Small cobbles of chert were imported to the site and were reduced into flakes by hard-hammer percussion. Cores were frequently rotated during knapping and the reduction strategy was ad hoc, lacking hierarchical reduction stages. Flake tools were used as-is or modified by retouching. Some flakes were selected for backing into geometric microliths, and backing techniques often reflected high levels of stoneworking skill to produce stylised scalene shapes. A review of contemporary archaeological evidence, and the context of the Saruq al-Hadid assemblage, suggest that microliths may have been made as stone armatures for arrows despite the contemporary use of copper-based arrowheads.

## Introduction

**Fig 1:**
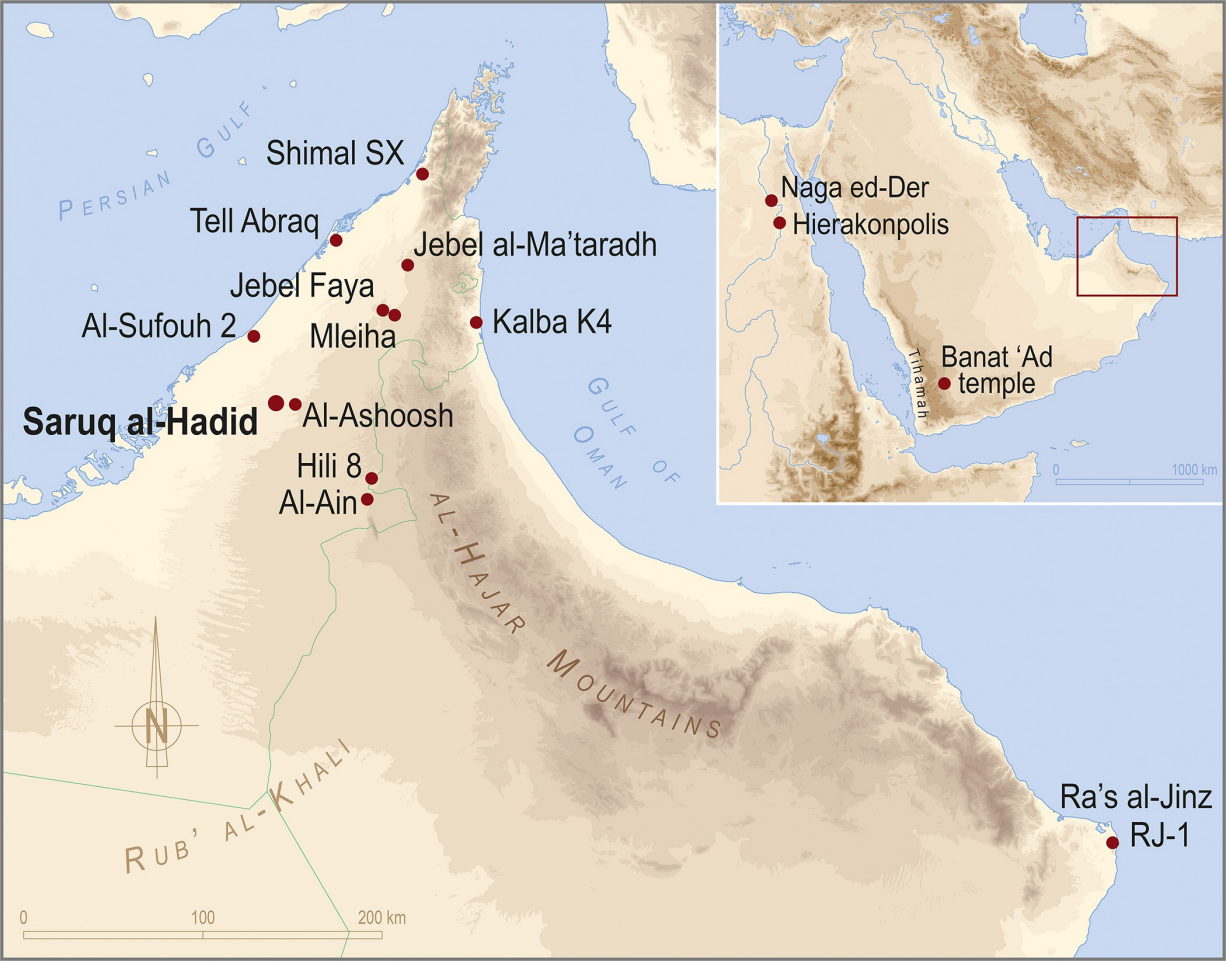
Southeastern Arabia showing locations discussed in the text. Figure by Hélène David-Cuny.

Saruq al-Hadid is a significant archaeological site located on the edge of the Rub’ al-Khali desert in Dubai, United Arab Emirates (Fig 1). The site consists of more than a square kilometer of discontinuous occupation deposits incorporated within dunes up to 6 metres deep, with complex assemblages of archaeological materials spanning the Neolithic to early Islamic periods, ca. 10,000–1,000 BP, and evidence for small-scale occupation continuing into recent centuries [1–6].

Recent excavations at Saruq al-Hadid recovered ca. 9100 stone artefacts, including backed microliths, from an extensive and dense midden deposit of animal bone which spans the Wadi Suq period and Late Bronze Age, c. 1750–1300 BC [6]. Here we describe the reduction sequence followed by the Wadi Suq/Late Bronze Age inhabitants of Saruq al-Hadid to produce stone tools, including backed microliths. We document a simple, low-cost and relatively low-skilled approach to core reduction that contrasts with high-skill techniques used to retouch flakes into microliths. The Saruq al-Hadid assemblage is contextualized against contemporary lithic assemblages from southeastern Arabia.

During this period at Saruq al-Hadid, stone tools were made and used alongside metal artefacts. The co-occurrence of stone and metal tools in the second millennium BCE is an important example of the continuity of stone technology after the development of metallurgy, a significant topic for lithic analysts [7–9] (e.g., [10–21]). Further, metal-age microlith technologies are largely unrecognized in popular explanations for the adoption of backed microlith technology in prehistory (e.g., [22]).

## The Saruq al-Hadid archaeological site

Saruq al-Hadid has witnessed intensive archaeological fieldwork since its discovery in 2002, including ongoing work by a range of local and international teams [1, 23–27]. From 2014 to 2017, the site was the focus of fieldwork by the Saruq al-Hadid Archaeological Research Project (SHARP) [5, 6], and the stone artefacts presented here derive from SHARP excavations.

Survey and excavation indicate that occupation at Saruq al-Hadid likely began as early as the eighth or seventh millennium BC, as suggested by the presence of diagnostic projectile points in deflated areas of the site (e.g., [28]: 35). This coincides with evidence for intensive Neolithic exploitation of the general area [3] during the Early to Mid-Holocene Humid Period, when grasslands and seasonal standing water are likely to have provided key resources for mobile herder-hunters [29, 30]. Hearth features excavated in the northern and western sectors of the site indicate continued occupation during subsequent periods of more variable but typically drier climate in the fourth and third millennia BC ([1]: Table 1), also attested at the nearby desert site of Al-Ashoosh [31]. Pit features and post holes dug into the gypsum pavement at Saruq al-Hadid comprise Horizon V, the earliest deposits excavated by SHARP. Material remains indicate that these deposits span the Umm an-Nar to early Wadi Suq transition, with modelled radiocarbon dates of ca. 2000–1750 BC [4].

**Table 1:** Counts of stone artefact types, Horizon IV, Saruq al-Hadid.

Artefact type	Context			Total
	1309	2008	Other	
Assayed stone	1	2	21	24
Core, freehand percussion	8	6	83	97
Core reduction flake, freehand percussion	220	170	30	420
Core, bipolar	--	--	3	3
Retouched flake	--	--	11	11
Tablet knife	--	--	11	11
Backed microlith, early stage	15	8	130	153
Backed microlith, final stage	1	6	87	94
Truncated piece	5	--	31	36
Truncation/backing flake	13	6	57	76
Recycled artefact	--	--	19	19
Hammerstone	1	--	4	5
Hammerstone spall	1	--	1	2
Stone crushing byproduct	3	8	--	11
Retouched ceramic	--	--	1	1
Unidentified reduction byproduct	15	12	1	28
Heat fracture	5	4	--	9
Manuport	1	2	8	11
Total	289	224	498	1011

**Fig 2:**
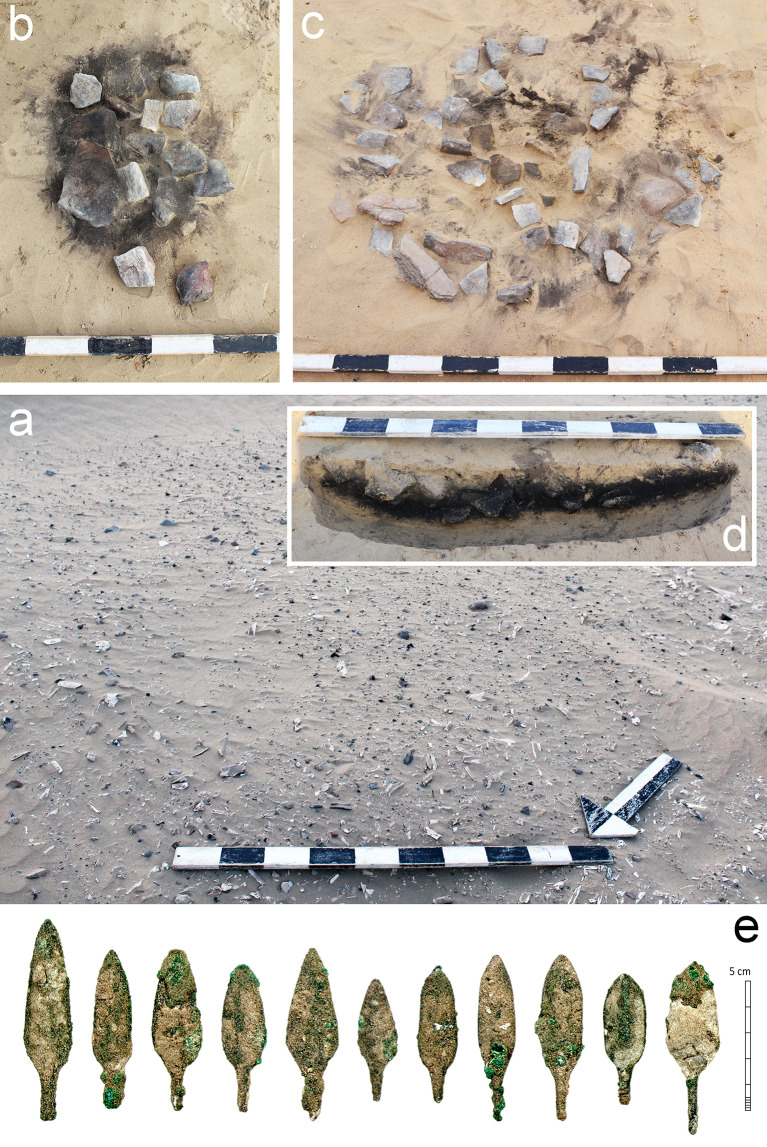
Cooking features and metal artefacts from the bone midden, Horizon IV, Saruq al-Hadid. (A) Bone midden prior to excavation. (B-D) Cooking features from contexts 2328 (B), 2332 (C), and 2338 (D). (E) Copper-base arrowheads from Horizon IV contexts (courtesy of Hélène David-Cuny). Scale bar 10 cm increments (A-D).

The stone artefact assemblage was recovered from a dense bone midden, labelled Horizon IV (Fig 2), which was deposited across the Wadi Suq period to Late Bronze Age transition, ca. 1750–1300 BC [4]. The bone layer measures at least 750 square metres and is up to 1 metre thick [5, 32, 33]. Midden excavations recovered more than one metric ton of bone fragments—primarily from wild animals, including oryx, camel, and gazelle—along with hearths, ceramics, marine shell, and soft stone vessels. Metal artefacts include 14 copper-based arrowheads of typical second millennium BC type (Fig 2E), which were found predominantly in the uppermost part of the midden. Stone-knapping debris is common throughout Horizon IV [34]. Although the deposits are partly deflated, the stone artefacts are in as-struck condition, with no evidence for patination, wind abrasion or other taphonomic damage. Analyses of excavated materials and absolute dating evidence demonstrate the high stratigraphic integrity of Horizon IV; thus, the stone assemblage is contextually secure [4, 35]. Occupation of Horizons V and IV corresponds to a period of arid climate which is broadly similar to the present-day environmental conditions in the region, and which follows a period of abrupt and intense aridification at the end of the third millennium BC [36].

Although dominated by the material residues of wild animal hunting, the bone midden at Saruq al-Hadid likely reflects a strategy of logistical mobility incorporating hunting and herd management, undertaken by members of a spatially dispersed, multi-sited community also engaged in agro-pastoral subsistence practices at sites outside the desert zone [5]. These activities prefigure the emergence of multi-community social gatherings, incorporating pilgrimage and associated cultic and craft activities, that effloresced at Saruq al-Hadid in the early Iron Age, ca. 1250–800 BC [5, 25, 26, 37, 38]. Occupation occurred intermittently from then until the sub-recent past, and encompassed a range of activities including periodic large-scale metal production [4].

## Lithic analysis methods and analytical sampling

All research work was undertaken under the auspices of contract 639/1/1413904 between the Dubai Municipality and the University of New England, Australia. All necessary permits were obtained for the described study, which complied with all relevant regulations. The Human Ethics Research Committee, University of New England, reviewed the project and confirmed in writing that ethics approval was not required.

The Saruq al-Hadid lithic analysis aimed to document the manufacturing techniques used to produce the stone tool assemblage recovered from Horizon IV. Methods follow the ‘reduction sequence’ approach, which involves classifying artefacts into technological types according to their inferred position in the reduction sequence model. In this approach, the structure behind stoneworking activities is reconstructed by identifying central tendencies in artefact morphology observed across the byproducts of multiple flaking events. The analytical process involves pattern-matching between artefacts in the archaeological assemblage and typologies of technologically-diagnostic byproducts of specific flaking techniques. The analyst examines the material results of flintknapping actions—cores and flakes and the negative scars on both—to reconstruct the core’s prior configuration and document its subsequent configuration. When repetitions of these configurations are discovered in an assemblage, the analyst infers that the repetitions reflect the flintknappers’ ‘plans of action’. Although stone reduction is a continuous process, the results are abstracted as a step-based ‘reduction sequence’, or *chaîne opératoire*. The inferences underpinning a reduction sequence are qualitative but have material consequences that can be explored through metrical analysis and tested through experiments. The approach and terminology in this analysis follows Moore [35, 39–41].

The stone artefact assemblage was recovered from 3 mm sieves or hand collected at the point of excavation. All stone artefacts from two contexts were analysed (1309 and 2008) (Table A in S1 Table), and partial analysis was conducted on 115 additional contexts. Partial analysis involved inspecting the material for ‘formed objects’ (stones with flakes removed from them, such as cores and retouched flakes), including backed microliths. A selection of these formed objects—and all of the identified microliths—were included in the analysis. The analytical sample (N = 1011) reflects about 11% of the recovered lithic assemblage (Table 1). All artefacts are stored at the Architectural Heritage and Archaeology Department, Dubai Municipality, Shindagha, Dubai, United Arab Emirates.

The most accurate reduction sequence models are inferred from sequences of conjoined artifacts—reassembled sets of sequentially-struck flakes—because they are an explicit record of actions applied by a flintknapper [39]. The spatial integrity of the Horizon IV assemblage was sufficiently good to allow some artefact conjoining, although this was not a focus of the study. Artefacts were grouped into ‘minimum analytical nodules’ (after [42]), referred to here as single reduction events (SREs), based on idiosyncratic colours or patterns in the chert. Some 47 SREs composed of 220 artefacts were identified across 18 excavation contexts, and most conjoins were identified within these SREs. The full analysis of context 1309 identified 21 SREs comprising 133 artefacts; full analysis of context 2008 identified 12 SREs comprising 38 artefacts. The largest SRE included 25 artefacts in context 1309.

## Results of the lithic analysis

### Stone sources and procurement

**Fig 3:**
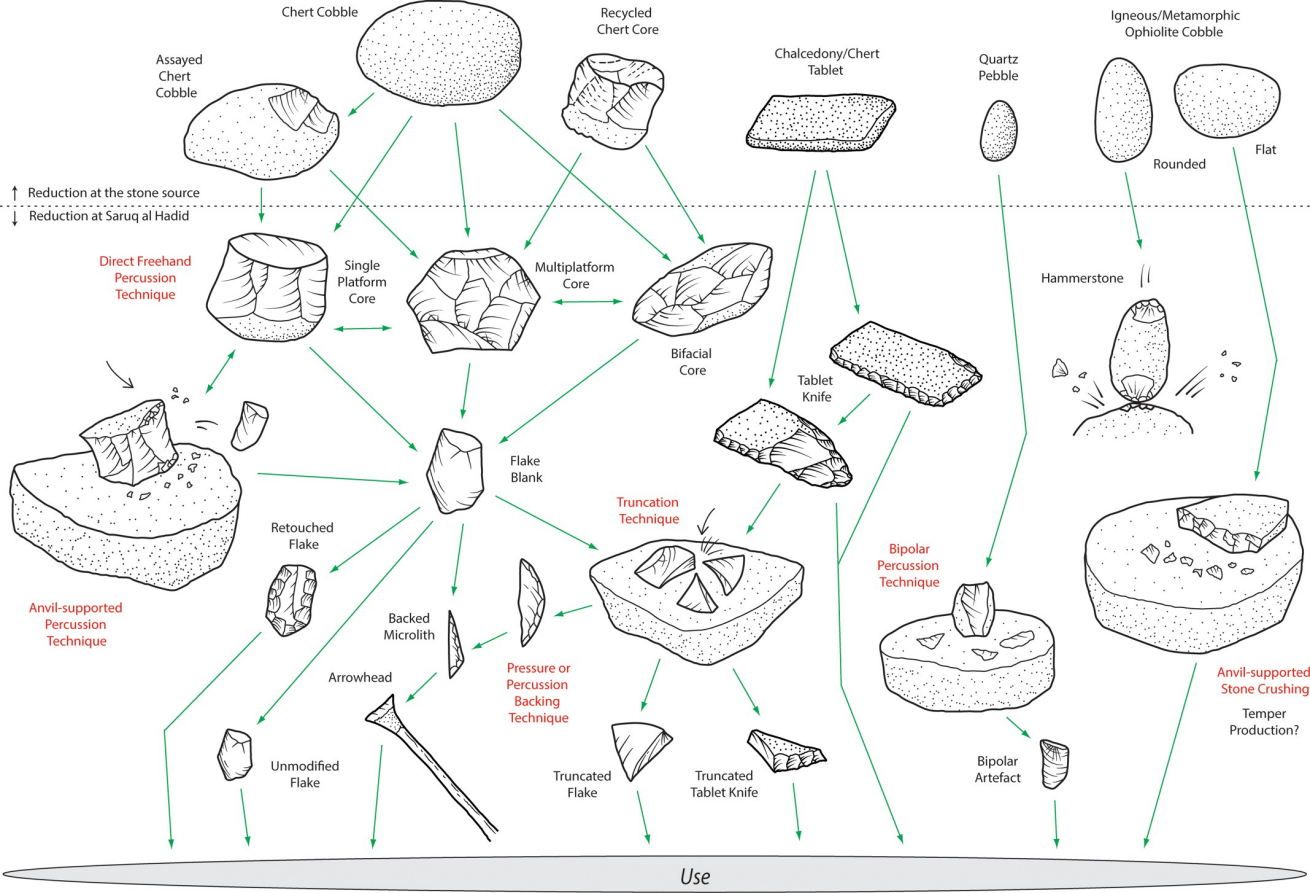
Stone reduction sequence model, Horizon IV, Saruq al-Hadid.

The Horizon IV reduction sequence mostly involved the reduction of small high-quality chert and chalcedony into sharp-edged flakes (Fig 3). Chert was procured elsewhere, carried to Saruq al-Hadid, and reduced on-site. These stones occur mostly as nodules, although thin tablets of high-quality chert and chalcedony were also exploited. The lithic resources of the region are under-researched, and geochemical characterisation of these outcrops is necessary, but the recurrence of distinctive colours, fossils, and patterns in some of the stone suggests that discrete sources may be identified with future research.

The raw materials in the assemblages were divided visually into five broad categories (Table B in S1 Table). Brown/tan chert is the most common, and is highly siliceous and of excellent flaking quality. Some brown/tan chert is banded with red stripes. According to Kallweit [43], this distinctive red-striped variant occurs along the mountains east of al-Ain. A flow-banded orange/yellow chert and chalcedony suggests hydrothermal silica deposition, perhaps as vugs or seams in bedrock. Black, white/grey, red, pink, and green chalcedony and chert are present in small amounts, including a distinctive white/gray chalcedony that outcrops in thin, cortex-covered tablets. Some of these stones, particularly the orange semi-transluscent chert, black chert, and green chalcedony, may come from the Jebel al-Ma’taradh source reported by Charpentier et al. [44] (MWM and LW, pers. obs. 2016). Stone types and colours in the flaked-stone assemblage are also seen in the stone bead assemblage at Saruq al-Hadid, particularly orange/yellow/red chalcedony (‘carnelian’), green chalcedony (‘green jade’), and yellow/red chert (‘jasper’) [45].

Some 30.4% of the analysed chert and chalcedony artefacts have cortical surfaces (Table C in S1 Table). Cortex is of three types: 1) soft, chalky surfaces; 2) hard, rounded, patinated surfaces, often with potlids; and 3) flat weathered surfaces exposed when stones broke apart along joints. Chalky cortex implies chert procurement at or near the bedrock source, as these surfaces disappear through sustained surface weathering. Hard cortex, in contrast, implies significant time on the surface, as is typical for lag deposits of stone in gibber pavements or secondary gravels. Joint separation at the stone source, through weathering or quarrying, can result in flaw line cortex on artefacts. Some artefacts displayed a mixture of both soft and hard cortex, indicating a near-bedrock source that has undergone some surface weathering, as is typical for colluvial slopes below eroding bedrock chert lenses. Most of the chert nodules in Horizon IV—particularly the brown/tan variety—were procured from the latter sources; these cobbles were marked by soft cortex. Brown/tan chert with soft cortex was noted in colluvial deposits of Jebel Faya near the modern town of Mleiha (MWM and LW, pers. obs. 2016), and this mountain range is a likely chert source area exploited by the Horizon IV flintknappers. In contrast, hard cortex dominates the orange/yellow chert and red chert, and white/gray chert comprises a mixture of cortex types.

**Fig 4:**
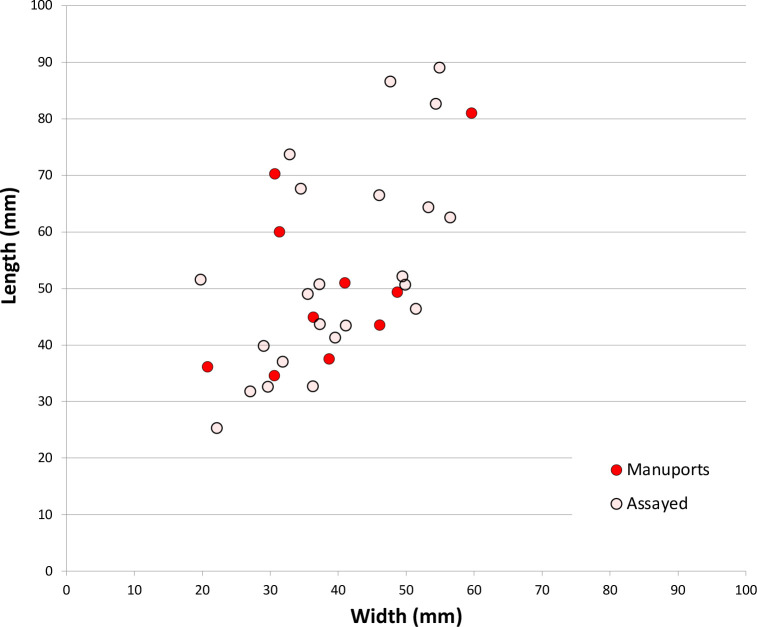
Scatterplot of unmodified and assayed chert cobble sizes, Horizon IV, Saruq al-Hadid. The unmodified chert cobbles were carried to the site (‘manuports’).

**Fig 5:**
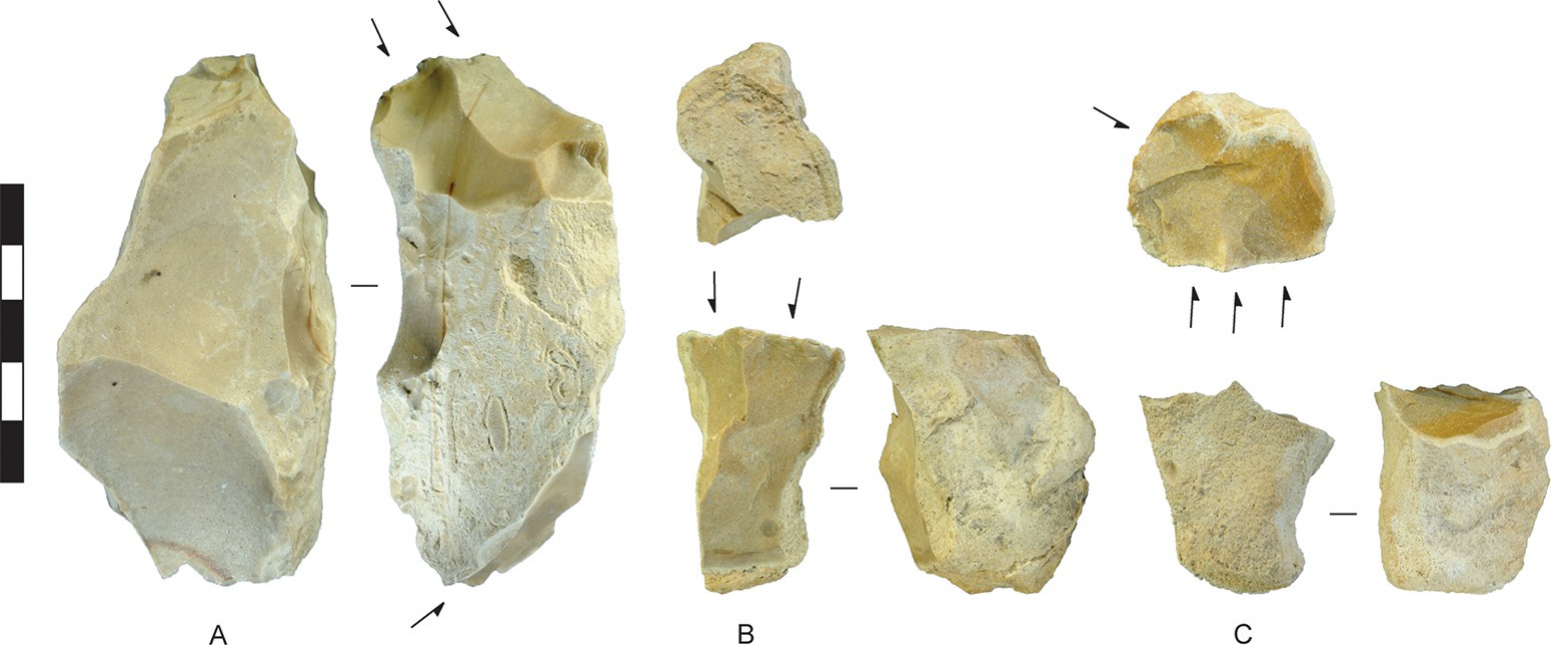
Assayed chert cobbles, Horizon IV, Saruq al-Hadid. Artefacts are from contexts 1748 (A), 1797 (B), and 2018 (C). Scale bar 50 mm.

Fig 4 shows the sizes of unmodified chert nodules brought to the site, and sizes of nodules assayed by the removal of less than four flakes. All are under 90 mm in maximum dimension and most are under 60 mm. Stones were probably assayed off-site, at the source, to check on the stone quality (after [46]), and transported to Saruq al-Hadid in a partially-reduced state, where some stones were discarded without further reduction (Fig 5). The stones are small, so transport effort would have been minimal.

**Fig 6:**
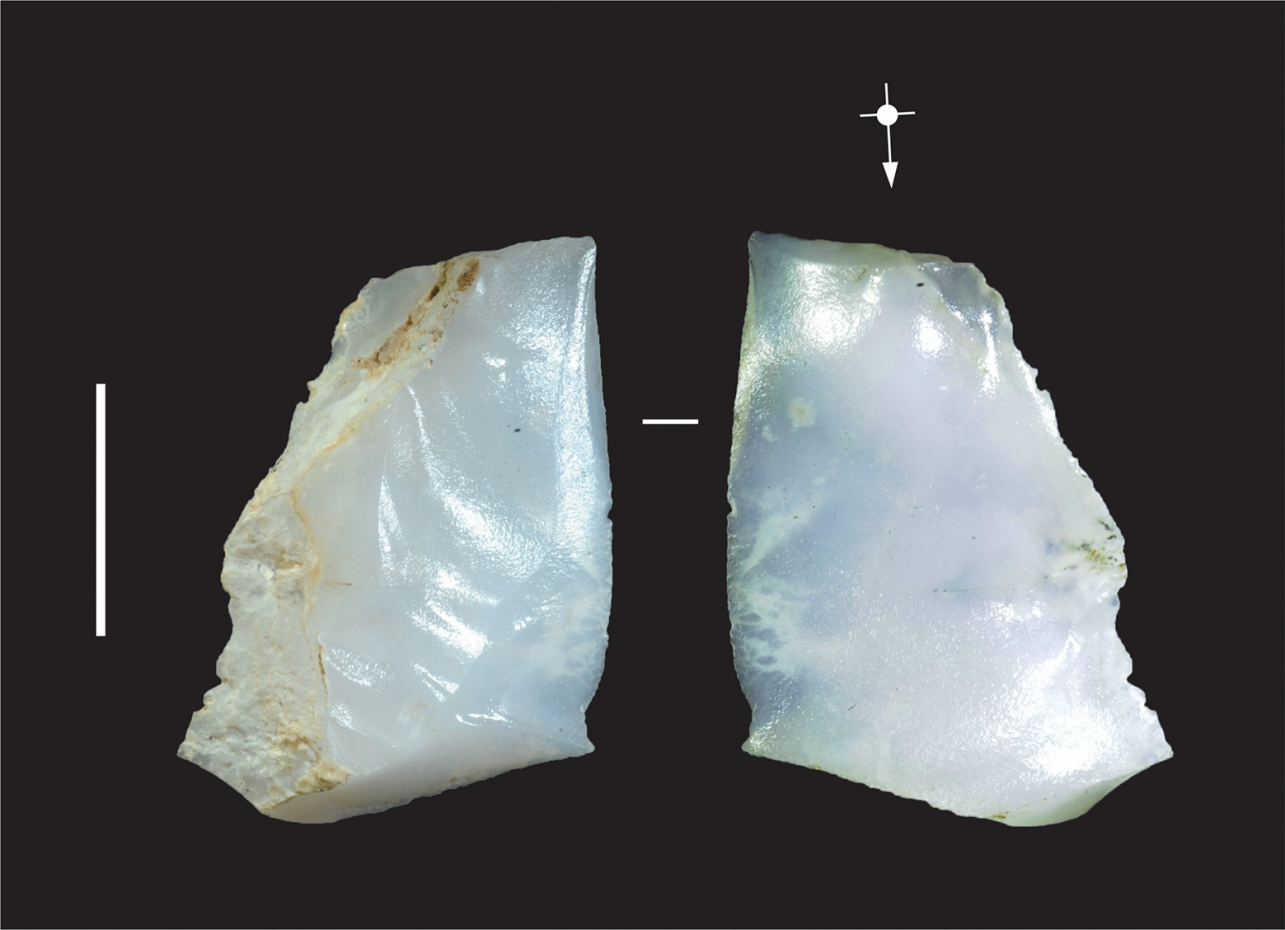
Chalcedony flake showing evidence for heat-treatment, Horizon IV, Saruq al-Hadid. Heat-treatment is suggested by the glossy surfaces. The dull surfaces are cortex. From context 2714. Scale bar 10 mm.

Heat-treatment is known to have been used in bead manufacture to alter the colour and lustre of iron-rich stones, and to substantially improve stone-flaking quality, but the practice has not been widely reported for Arabian flaked-stone assemblages. However, ten artefacts were identified at Saruq al-Hadid that may have been altered by heat-treatment. Controlled heat-treatment can markedly reduce the tensile strength of siliceous stones, making them easier to flake, but the resulting tools are relatively more brittle than those made from untreated stone. Heat-treated stone has glossy, lustrous flake scars, and chalcedony alters from a fibrous texture to a smooth, glossy surface [47]. Heat-treatment can also enhance the red hues of iron-rich stones. Our experimental heat-treatment of chert samples from Jebel Faya and Jebel al-Ma’taradh did not result in a dramatic colour change, but the process substantially increased the stones’ glossiness, particularly the chalcedony samples. The flake scars on a stone created prior to heat-treatment retain the original matte texture, but post-heat-treatment scars are glossy. Heat-treatment is reliably indicated by artefacts with ‘differential gloss’ from pre- and post-heat-treatment reduction. The ten Saruq al-Hadid artefacts identified as possibly heat-treated demonstrated highly glossy textures (Fig 6), but not differential gloss between flake scars. Although the Saruq al-Hadid flintknappers occasionally heat-treated their raw materials, this was not a regular step in the reduction sequence.

**Fig 7:**
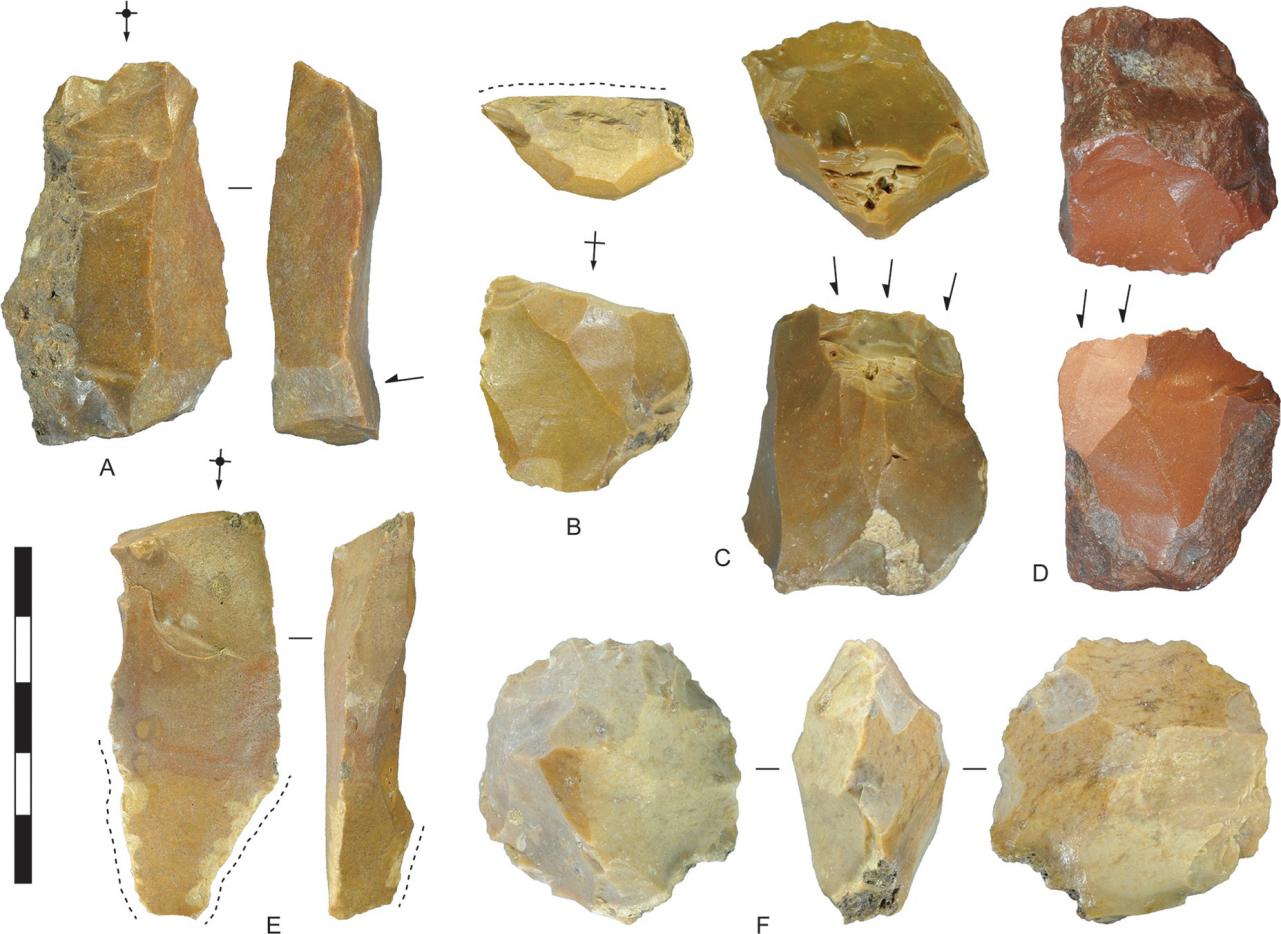
Chert artefacts recycled from earlier lithic scatters and further reduced or utilised, Horizon IV, Saruq al-Hadid. Reworking is indicated by relatively fresh flake scars intruding into patinated flake scars. (A) Blade-like flake with more recent unifacial reduction at the distal end. (B) Distal end of a flake with more recent steep unifacial retouch across the proximal end. The retouching is indicated by a dashed line. (C) Single-platform core with crushed platform from a more recent attempt at removing a flake down the core face. (D) Bifacial core with a more recent flake struck from the same platform edge. (E) Blade-like flake recycled as a tool. More recent use-wear flaking is present on the lateral edges and dorsal ridge, indicated by the dashed lines. (F) Bifacial centripetal core with older, patinated scar remnants on both faces. From contexts 1794 (A), 2021 (B), 2012 (C), 2019 (D), 1790 (E), and 4802 (F). Scale bar 50 mm.

Some 17 chert and 2 chalcedony artefacts display flake scars with differential weathering, with older scars intruded by fresh scars. The older scars are mechanically and/or chemically weathered, resulting in a slightly ‘polished’ appearance and darker value than the more recent scars (Fig 7). This indicates that Wadi Suq/Late Bronze Age flintknappers sometimes collected cores discarded during earlier stone-flaking activities, possibly in the Neolithic or earlier, and carried them to the midden for further reduction. Bronze Age scavenging evidently altered the nature of Neolithic-age lithic scatters by the removal of cores and larger flakes. Casana et al. [3] report extensive Neolithic lithic scatters within ten kilometres of Saruq al-Hadid, and this may have been the closest chert source for the Wadi Suq/Late Bronze Age flintknappers. Material recycling (see [48]) was a persistent behaviour at Saruq al-Hadid: soft stone from Wadi Suq/Late Bronze Age deposits was recycled during the Iron Age [49], and on-site Iron Age and post-Iron Age metallurgical practices also involved substantial recycling [50].

**Fig 8:**
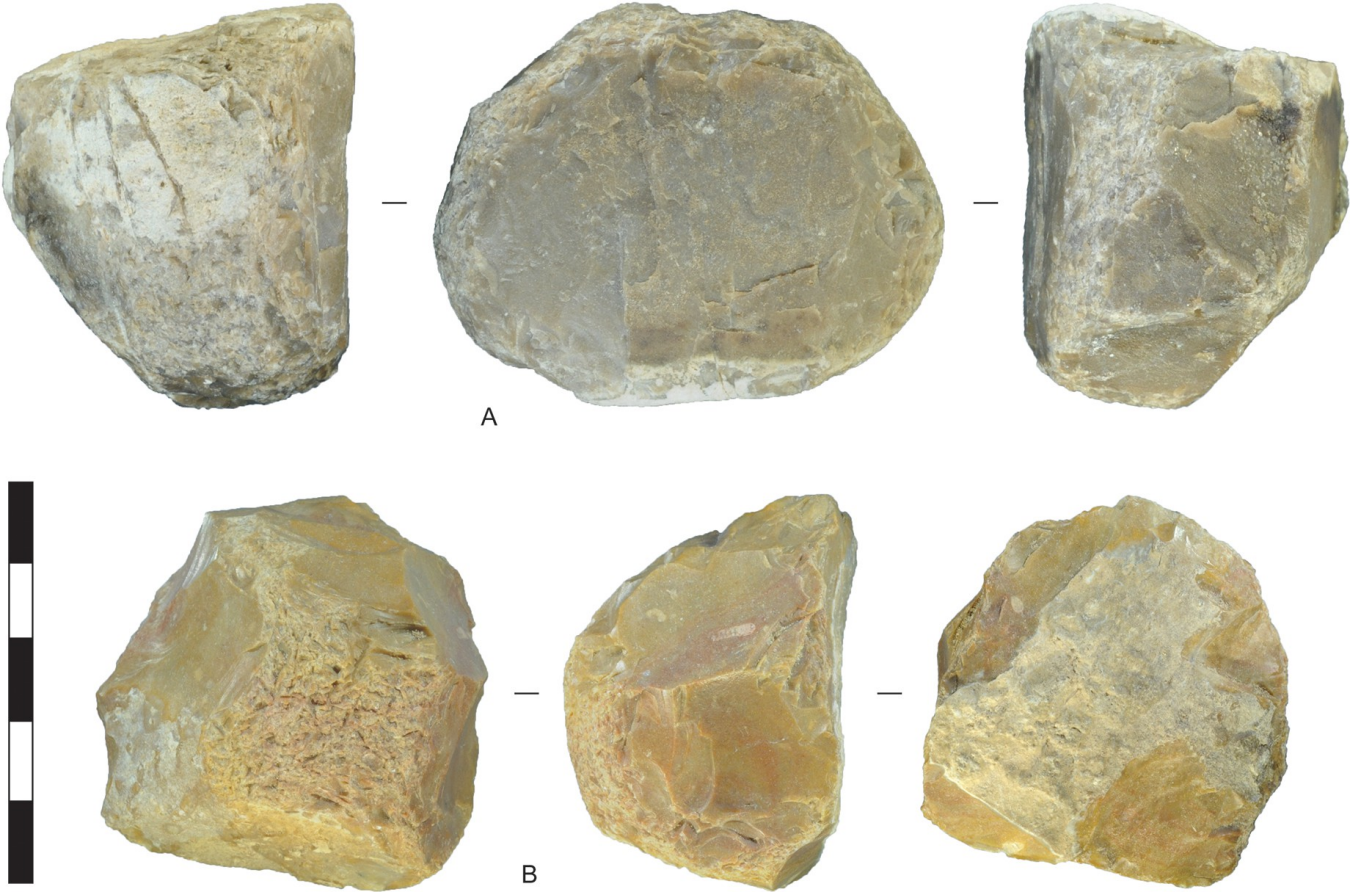
Hammerstones, Horizon IV, Saruq al-Hadid. (A) Dolomite hammerstone with wear facets around the perimeter. (B) Chert hammerstone with a wear facet on one face. The hammerstone was shaped by percussion flaking prior to use as a hammer. From contexts 2446 (A) and 2006 (B). Scale bar 50 mm.

**Fig 9:**
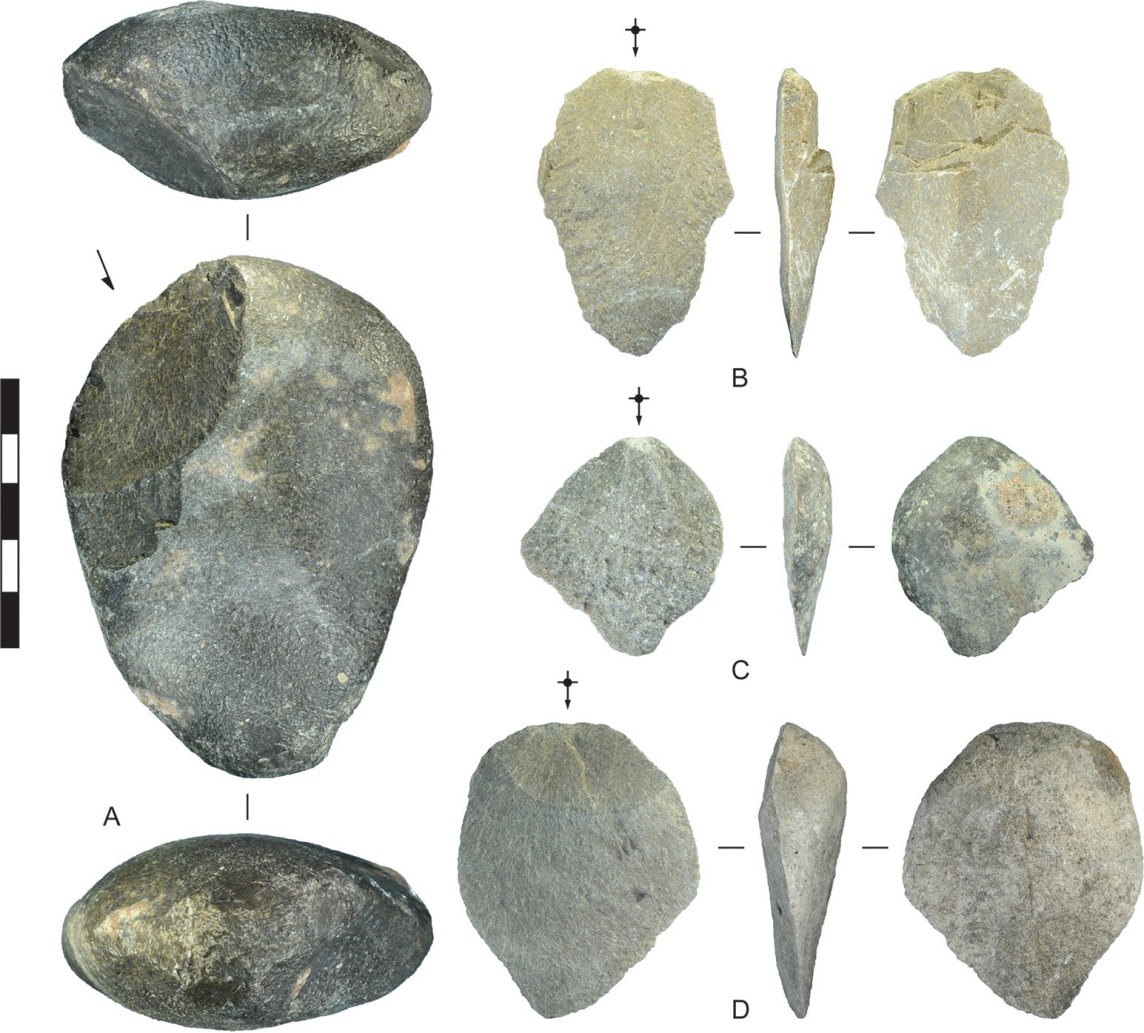
Hammerstones and flakes, Horizon IV, Saruq al-Hadid. (A) Dolomite cobble with hammerstone wear and a use-wear scar resulting from a heavy percussion blow (arrow). B) Dolerite flake initiated by wedging. The prior negative scars suggest that this flake was produced in the reduction of a dolerite core, rather than from use as a hammerstone. C) Dolomite flake initiated by wedging. D) Dolomite flake initiated by wedging. From contexts 1293 (A), 2117 (B), 2315 (C), and 2325 (D). Scale bar 50 mm.

Metamorphic stone cobbles were used for pounding and flaking tasks in Horizon IV, producing flakes from direct percussion and detritus from percussion activities, such as hammerstones and hammerstone spalls (Figs 8 and 9). Rounded metamorphic cobbles are available as widespread lag deposits of the Semail Ophiolite [51], including local gravels of the Barzaman Formation in deflated zones between dunes. Some of these cobbles are relatively large, ranging up to 150 mm in largest dimension. They likely relate in part to the manufacture and use of grinding stones in Horizon IV. One metamorphic cobble-flake has percussion pitting on the platform area that occurred prior to detachment, and is likely a hammerstone spall. Other cobble-flakes may also be hammerstone spalls (e.g., Fig 9B–9D), but lack pitting and were therefore classified as flakes. The analysis of contexts 1309 and 2008 show that although metamorphic stones and debris compose 10.6% of the flaked-stone assemblage by count, they compose 26.5% by weight. Not included in this total are large numbers of hearth heat-retainer stones (i.e., fire-broken rock) and grinding stones and fragments, which indicate that considerable effort was expended in procuring and transporting metamorphic stones.

Four small, flat metamorphic stone fragments were identified in contexts 1309 and 2008 that show evidence of backing-like fractures through their thickness, and crushing along the platform edges. Two of these pieces are flakes struck by hard-hammer percussion, and the other two are platy cobble sections. These objects are made from coarse, poorly-silicified materials, and seem unsuitable for tools. They may be byproducts from smashing thin, flat stones into temper for ceramic production. Seven small backing-like ‘crushing flakes’ were also identified that probably relate to this process (Table D in S1 Table). If this identification is correct, it suggests that ceramics were being produced on-site using ophiolitic materials from local lag deposits as temper.

**Fig 10:**
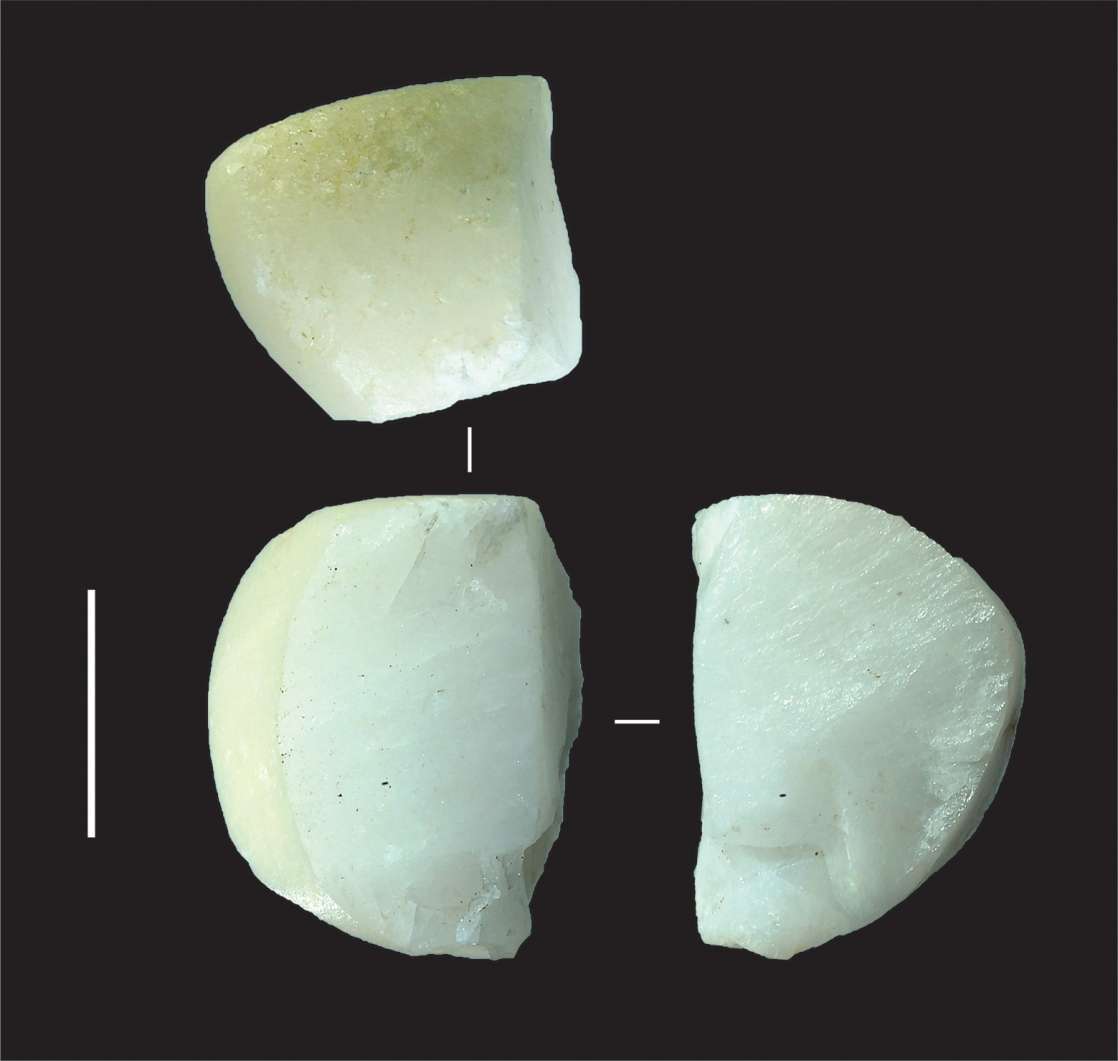
Bipolar core made on a quartz pebble, Horizon IV, Saruq al-Hadid. The artefact is from context 1797. Scale bar 10 mm.

Other materials flaked at the site include one core and four flakes of milky quartz. One of these artefacts is a small fluvially-rounded milky quartz pebble reduced by bipolar percussion (Fig 10). The quartz may have also been procured from local ophiolite. One ceramic fragment was identified with steep unifacial retouching on one edge, perhaps for use as a tool or from recycling for temper (grog) production. One percussion flake of shell, probably marine in origin [33], and identified by chance in a lithic debitage bag, provides further evidence for Weeks et al.’s ([52]: 14) suggestion that early stages of shell object manufacture occurred at Saruq al-Hadid.

If the stone source regions identified above are correct, it indicates that highly siliceous stones were brought to Saruq al-Hadid from stone sources in several directions and up to 60 to 100 kilometres away. Long-distance movement of soft-stone vessels [49], pottery [37], stone beads [45], and marine shell [33, 52] also occurred during this period. The long-distance transport of flaked stone was augmented with local stones from ophiolite lag deposits between dunes, and chert cores from earlier lithic scatters in the vicinity of the site.

### Core and flake assemblage

**Fig 11:**
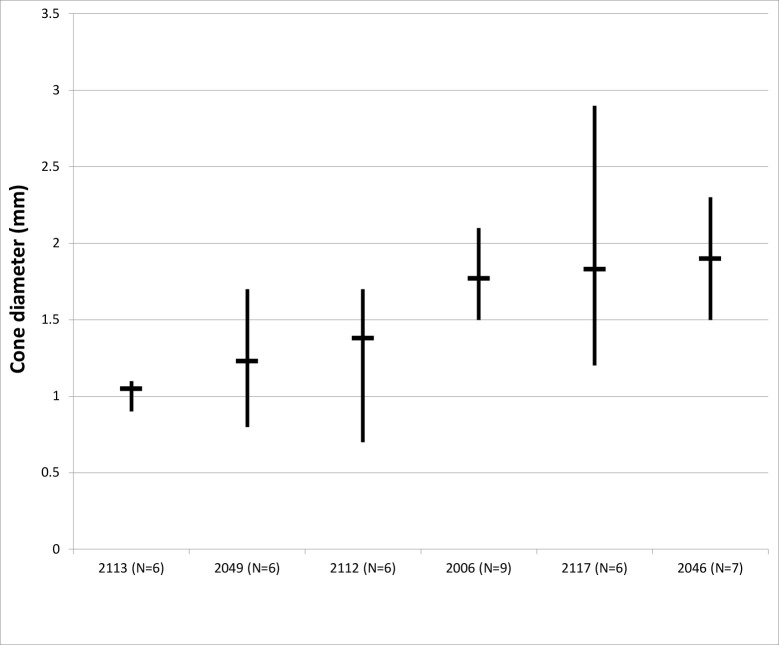
Chart showing the mean and range of incipient cone diameters on six cores, listed by excavation context, Horizon IV, Saruq al-Hadid.

The majority of knapping at Saruq al-Hadid involved flake-making by freehand hard-hammer percussion. Ring cracks from unsuccessful hard-hammer percussion blows are preserved on some cores. When struck by the indentor, the stone under the contact circle is under compression, causing the molecules to be ‘drawn inward by the indentor like the membrane of a drum’ ([53]: 68). This creates a zone of tensile stress around the perimeter of the zone of compression. Siliceous stone is weaker in tension than in compression, and the circular ring crack forms at the boundary of the compressed area ([54]: 685). The diameters of ring cracks were measured on 13 cores using a 0.10 mm-increment scale bar insert into a 10x Eschenbach hand lens. The ring cracks average 1.6 ± 0.53 mm diameter (N = 55), ranging from 0.7 to 2.9 mm (Fig 11). A ring crack is about 12% larger than the contact surface with the indentor ([55] in [56]: 42), meaning that the zones of contact on the Saruq al-Hadid cores averaged about 1.4 mm diameter, and not more than 2.5 mm diameter. This, in turn, suggests that indentors for flake-making possessed defined working ends that concentrated the percussion force within a very small contact zone.

The hammerstones in Figs 8 and 9 have broad working faces, and the ring-crack data suggests that it is unlikely that they were used to reduce the chert cores, but no hammers with defined working ends were recovered in the assemblage. It may be that the hammerstones we recorded were discarded by their users after the defined working surfaces were rounded by stone-flaking attrition, but they may also have been used for hammer-dressing in manufacturing ground-stone tools or processing animal bone for marrow extraction (e.g., [57])—tasks that do not require as carefully-targeted blows as for controlled stone-flaking. Alternatively, copper-based indentors may have been used as percussion tools, but metal smears (e.g., [58]) were not identified on flake or core platforms at Saruq al-Hadid.

Core reduction produced three types of cores: single-platform, multiplatform, and bifacial. Single-platform cores were reduced from one platform surface, multiplatform cores were reduced from multiple platform surfaces, and bifacial cores were reduced to two faces from a centripetal platform edge. The angle between the platform surface and core face approaches 85–90 degrees on single-platform and multiplatform cores, but is more acute on bifacial cores. Multiplatform cores began as single platform cores, but were rotated during reduction and a new platform surface was established. Most multiplatform cores (67%) show evidence of one rotation, although some show evidence of 2 or 3 rotations (Table 2).

**Table 2:** Number of platforms on cores, all contexts, Horizon IV, Saruq al-Hadid.

Core type	Number of platforms				Total
	1 (not rotated)	2 (1 rotation)	3 (2 rotations)	4 (3 rotations)	
Single platform	28	--	--	--	28
Multiplatform	--	37	16	2	55
Bifacial centripetal	11	3	--	--	14
Total	39	40	16	2	97

Scars created early in reduction are wholly or partly ‘erased’ (after [59]) by later reduction, and the cores discarded at Saruq al-Hadid mostly preserve the flake scars from the reduction immediately preceding discard. For this reason, the evidence for core rotation preserved on cores may underestimate the frequency of rotation because earlier platforms are erased. One way to gauge this is by recording the abundance of ‘redirecting’ flakes in an assemblage. A redirecting flake preserves a former platform edge on the dorsal surface, and it documents the erasure of a previous platform edge following core rotation. The frequencies of redirecting flakes range from 3.6% to 5.9% of the core reduction flakes from the two analysed contexts, and cores from those assemblages document from 1 to 2 rotations (Table E in S1 Table).

The average number of scars still extant on single platform cores at the time of discard is 9.8 ± 4.4 (N = 19). Using this as a proxy of the average number of flakes removed from a core prior to rotation, we can estimate that one redirecting flake might be produced for roughly every 10 core reduction flakes. Applying this estimate to the contexts with complete artefact counts, we can predict the presence of 22 redirecting flakes in the assemblage of 220 flakes from context 1309, and 17 redirecting flakes in the assemblage of 170 core reduction flakes from context 2008. The actual redirecting flake count for context 1309 (N = 8) is 64% lower than predicted, and the actual count for context 2008 is 54% (N = 10) lower than predicted. This lower-than-expected count is likely the result of flake scar erasure.

**Fig 12:**
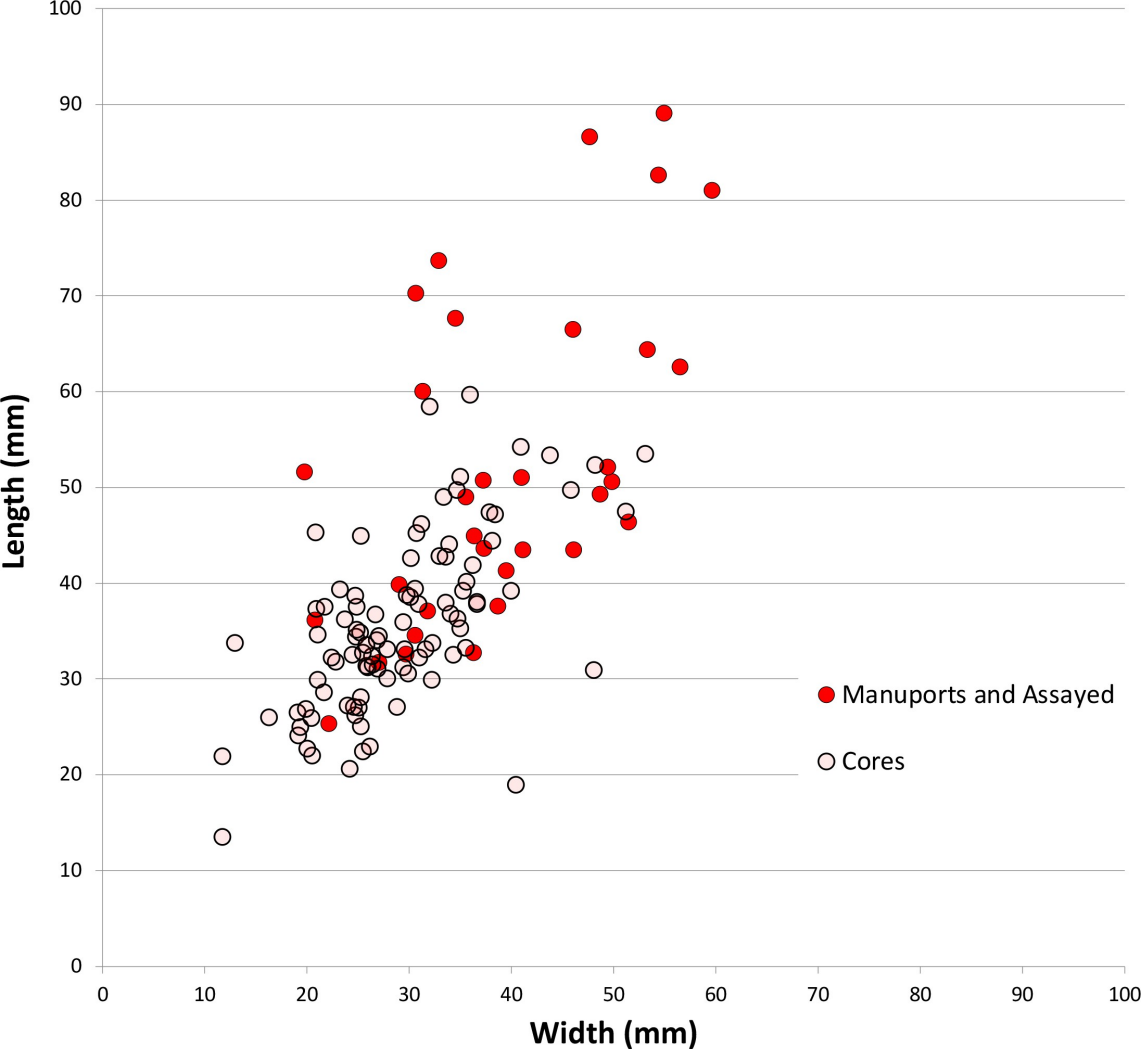
Scatterplot comparing core sizes to manuport/assayed chert cobble sizes, Horizon IV, Saruq al-Hadid.

The discarded cores are small, measuring 36.3 ± 8.9 mm (N = 94) in maximum dimension, on average. Unmodified chert manuports and assayed cobbles abandoned at the site are larger on average than the discarded cores (51.9 ± 16.4 mm, N = 32), and the difference in averages suggest that stone dimensions decreased by 30% through reduction (Fig 12). When the same calculation is done by weight rather than maximum dimension, about 46% of stone mass was lost from the manuport/assayed cobble phase (56.7 ± 38.9 g, N = 32) to the cores discarded into the deposits (30.9 ± 20.8 g, N = 94). Extrapolating to the flake assemblage, the chert/chalcedony core reduction flakes in context 1309 (N = 217) weigh 423 g in total, and in context 2009 the core reduction flakes (N = 166) weigh 249 g in total. Using the 46% attrition inferred above, these weights suggest the reduction of 16 cores in context 1309 and 10 cores in context 2009; the actual number of discarded cores in these contexts was 8 and 6 respectively, about half the predicted number in both cases. These calculations should be interpreted cautiously because the standard deviations on the metrics are relatively large, and using average dimensions masks this variability. The calculations also assume that the manuports and assayed cobbles are reasonable proxies of the sizes of stones brought into the site. The calculations do, however, provide broad parameters for assessing the amount of chert/chalcedony imported and the amount of stone reduction occurring there. The stone flakes discarded in contexts 1309 and 2009 did not clearly derive from the cores discarded in those locations, based on colour or SREs, so cores were not always discarded where they were reduced.

**Fig 13:**
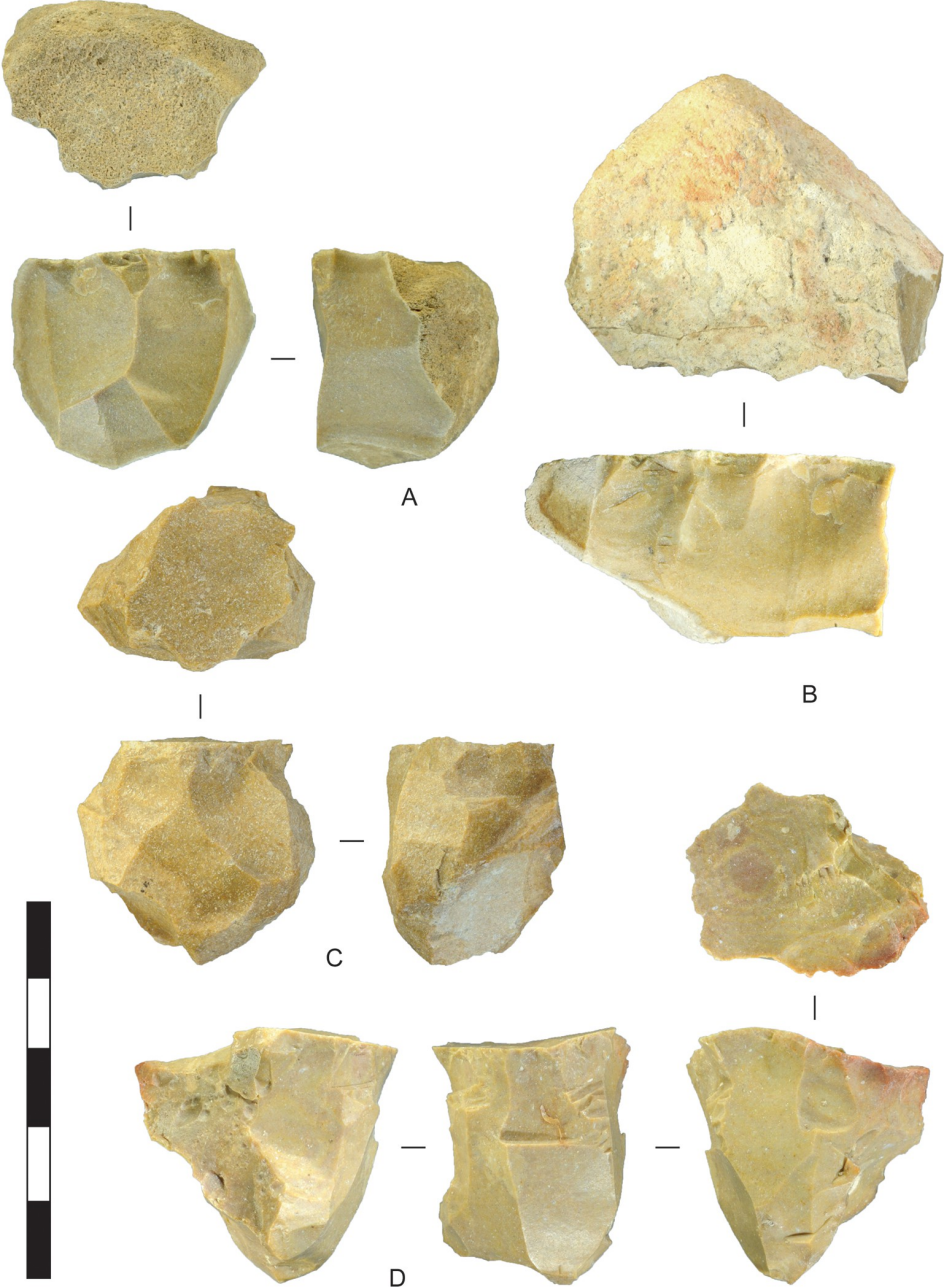
Chert single platform cores, Horizon IV, Saruq al-Hadid. (A, B) Cores reduced around part of the perimeter from cortical platform surfaces. (C, D) Cores reduced around most of the perimeter from a platform composed of a large flake scar. From contexts 1794 (A), 1797 (B), 2018 (C), and 2016 (D). Scale bar 50 mm.

**Fig 14:**
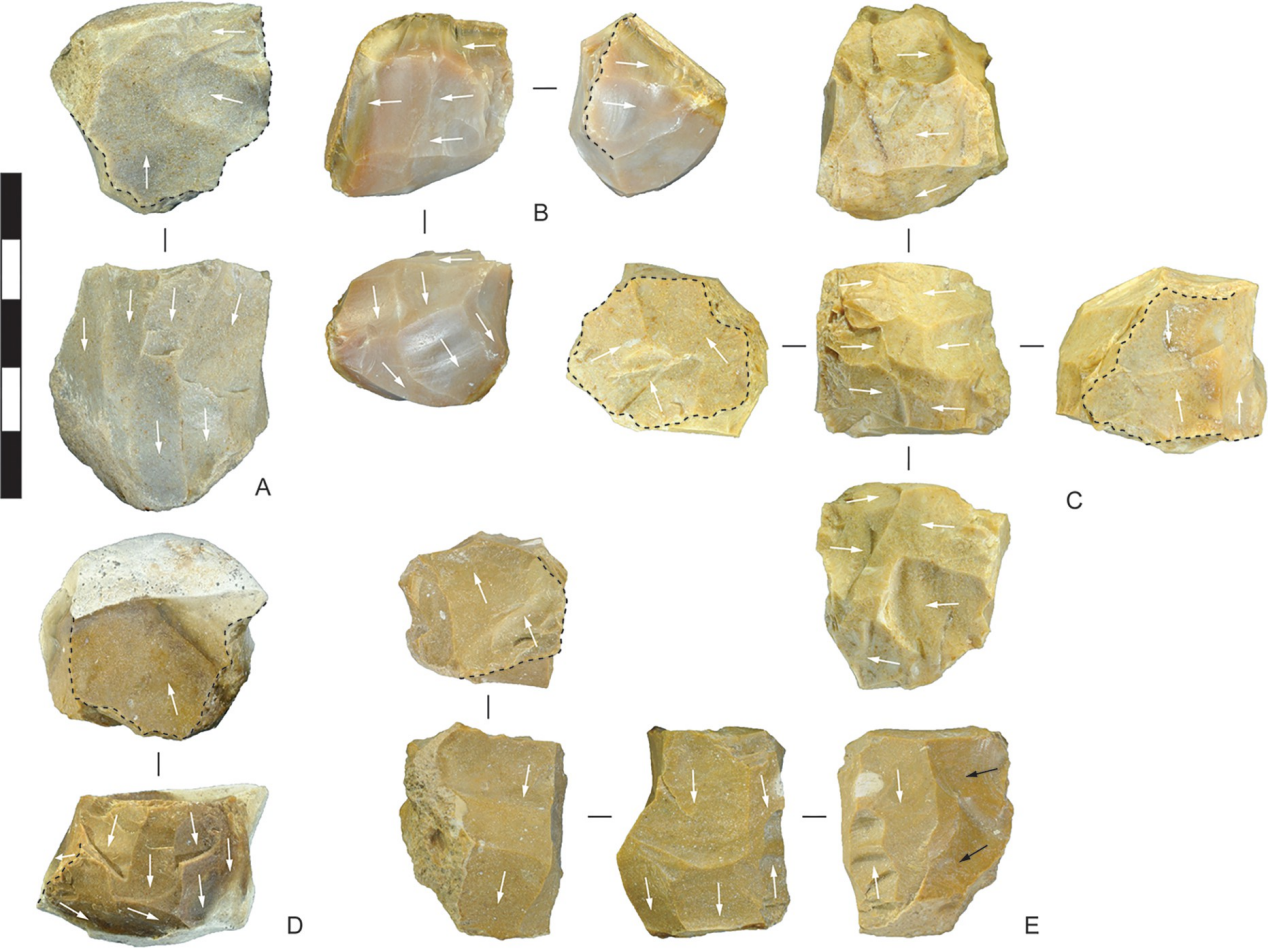
Chert single platform and multiplatform cores, Horizon IV, Saruq al-Hadid. Dotted black lines outline platform surfaces, and arrows show the direction of major flake scars. (A) Core with three flakes removed across the end followed by rotation and striking flakes down the adjacent face using the prior scars as the platform. The reduction surfaces are adjacent to the one platform edge, so this is classified as a single platform core. (B) Core reduced in a similar way to A, but with evidence for two prior rotations and reduction from platforms that no longer exist. The final platform was established on the lateral margin of a prior scar. Only one platform now exists, so this is classified as a single platform core. (C) Core with a ‘cuboidal’ morphology, with bidirectional reduction down the core face from platforms at either end. Two independent platforms exist, so this is classified as a multiplatform core. (D) Core reduced in a similar way to A, but from a platform surface composed of a single large negative scar. The core was rotated and flakes were struck from the lateral margin of a prior scar, creating a second independent platform. The core is classified as multiplatform. (E) Core reduced bidirectionally from a multi-scar platform at one end (similar to A) and a cortical platform at the opposite end. The core has two independent platforms and is classified as multiplatform. The blank was a core recycled from an older lithic scatter; the black arrows indicate scars from the earlier reduction. All of these cores have platforms oriented at close to 90 degrees to the core face, and platforms on A-D are crushed, perhaps indicating anvil support. From contexts 2231 (A), 2111 (B), 1309 (C), 2823 (D), and 2325 (E). Scale bar 50 mm.

The core reduction sequence for single-platform and multiplatform cores (Figs 13 and 14) began with assaying. In the case of single-platform cores with cortical platforms, the configuration established in assaying continued until the stone was substantially reduced and discarded (e.g., Fig 13A and 13B). The cobble axis selected for flake production was not always the longest one (e.g., Fig 13B). Sometimes the core was turned after assaying and those negative scars were used as a platform for striking flakes down an adjacent face. Fig 5C shows an assayed cobble with the initial flake-scar platform established, but the core was abandoned at that point. Fig 13C and 13D are cores where the flake-scar platform was used to strike flakes from around most of the perimeters of the cores. Of the 28 single-platform cores analysed from Horizon IV, 57% were reduced from flake-scar platforms. This compares to 54% of chert/chalcedony core reduction flakes struck from single-facet flake-scar platforms (excluding edge and collapsed platforms, Table 3). A surprisingly high proportion of cobbles—46%—were reduced entirely from cortical platforms. Cores were sometimes rotated to cortical platforms after the earlier platform(s) were abandoned (e.g, Fig 14E), accounting for some of the cortical-platform flakes. If the preceding platforms were entirely erased by core rotation, and the discarded core only had one platform remaining, it was classified as a single-platform core, and this typological convention results in an overestimation of the single-platform reduction strategy.

**Table 3:** Platform types on core reduction flakes, Horizon IV, Saruq al-Hadid.

Platform type ^1^	Context		Total
	1309	2008	
Cortical ^2^	61	47	108
Single facet ^3^	67	47	114
Dihedral ^4^	4	3	7
Multifacet ^5^	3	3	6
Edge ^6^	11	7	18
Collapsed ^7^	18	10	28
Total	164	117	281

^1^ For conchoidally-initiated flakes, platform types were defined by the nature of the surface bearing the point of force application (PFA). For bend-initiated flakes, which lack a PFA, classification was based on the number of scars at the platform’s middle.

^2^ The PFA is on a cortical surface.

^3^ The PFA is on a negative scar surface.

^4^ The PFA is on an arris between two flake scars.

^5^ The PFA is on a surface with multiple flake scars.

^6^ The PFA is directly on the platform edge.

^7^ The PFA is eliminated by crushing.

The new platform on a rotated (multiplatform) core was usually the lateral side (e.g., Fig 14B and 14D) or distal end of a flake scar created from a preceding platform. Often the core was rotated and flakes were struck across the base of the core, usually from the distal end of a flake scar, establishing a platform surface for striking flakes in a direction opposite to the flakes struck from the preceding platform. This created bidirectional cores with a roughly cuboidal morphology (e.g., Fig 14C and 14D). Another variant appears to have involved alternating the producer face and platform face of the core; that is, the platform face became, later in reduction, the producer face, and the proximal ends of the former producer scars became the new platform. This alternating pattern continued until the core was discarded (e.g., Fig 14A).

**Fig 15:**
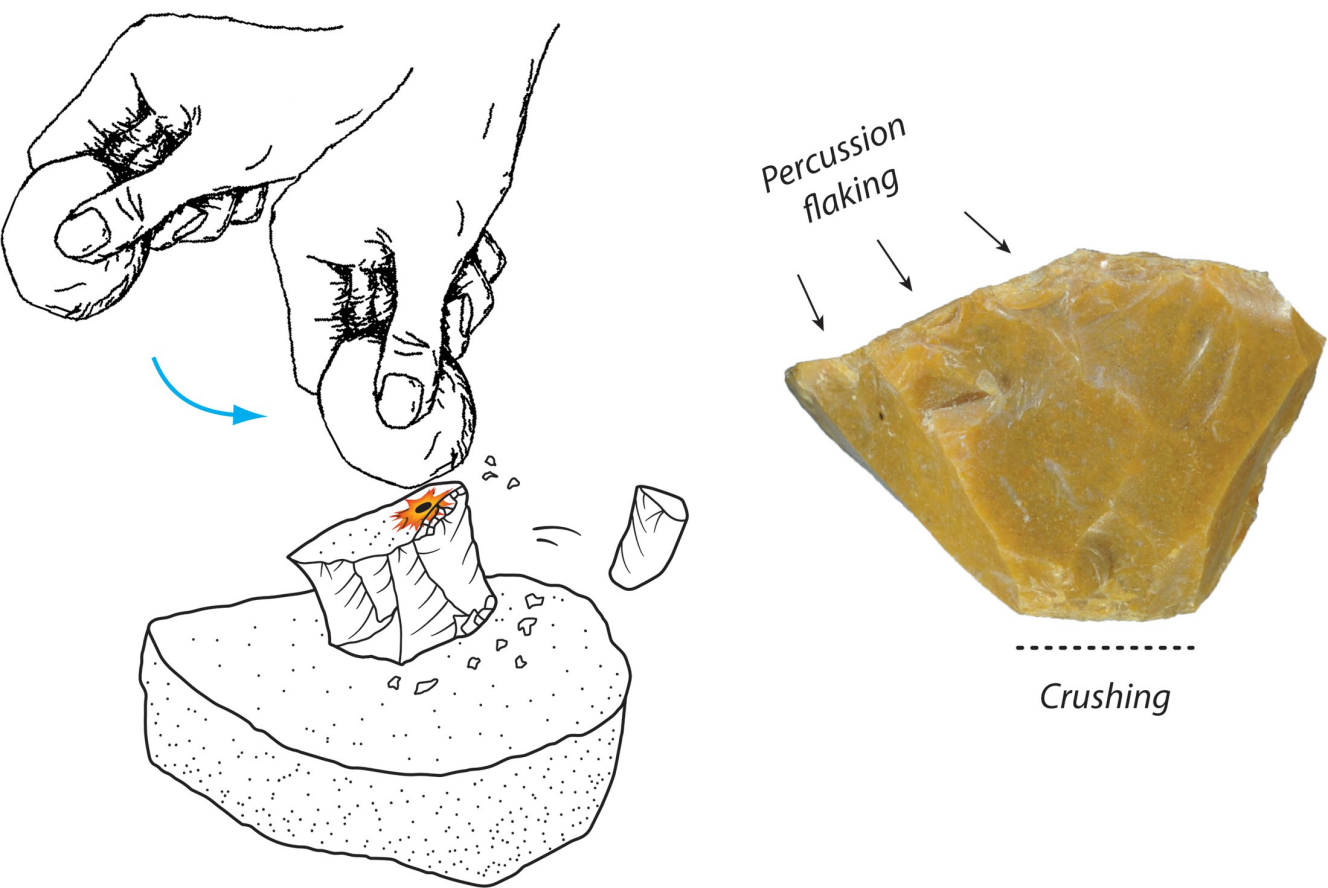
Schematic representation of anvil-supported direct percussion, Horizon IV, Saruq al-Hadid. The chert multiplatform core is from context 1797.

Some of the cores show pronounced unifacial or bifacial crushing on platform edges. The crushing is often along much of the platform edge and is inconsistent with platform collapse from individual blows or overhang removal down the core face. Rather, the crushing suggests that the technique for removing flakes from these small cores may have sometimes been augmented by distal core support on a hard anvil (Fig 15). Movement of the core on the anvil created the crushing. The blows were oblique to the core mass, rather than straight-in as with bipolar reduction and truncation/backing (discussed below). Striking flakes in this may have involved a technique where the knapper swung the hammer across the platform of the core, almost parallel to the plane of the anvil support. This technique helps maintain a very steep platform angle, and the support decreases the frequency of overstrikes through the end of the core. Flakes with overstruck terminations are rare in the flake assemblage.

**Fig 16:**
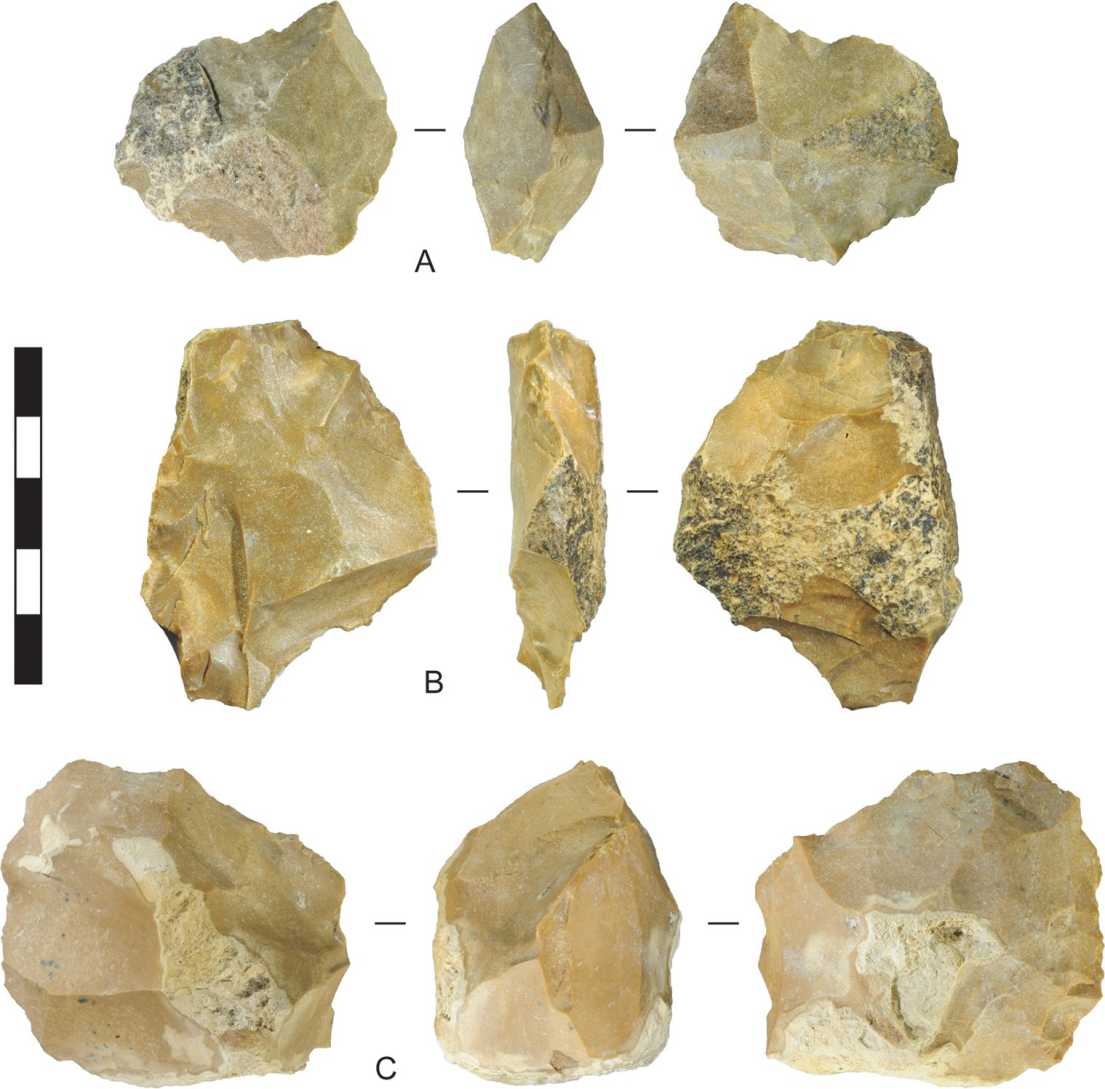
Chert bifacial centripetal cores, Horizon IV, Saruq al-Hadid. The artefacts are from contexts 2018 (A), 1790 (B), and 2022 (C). Scale bar 50 mm.

**Fig 17:**
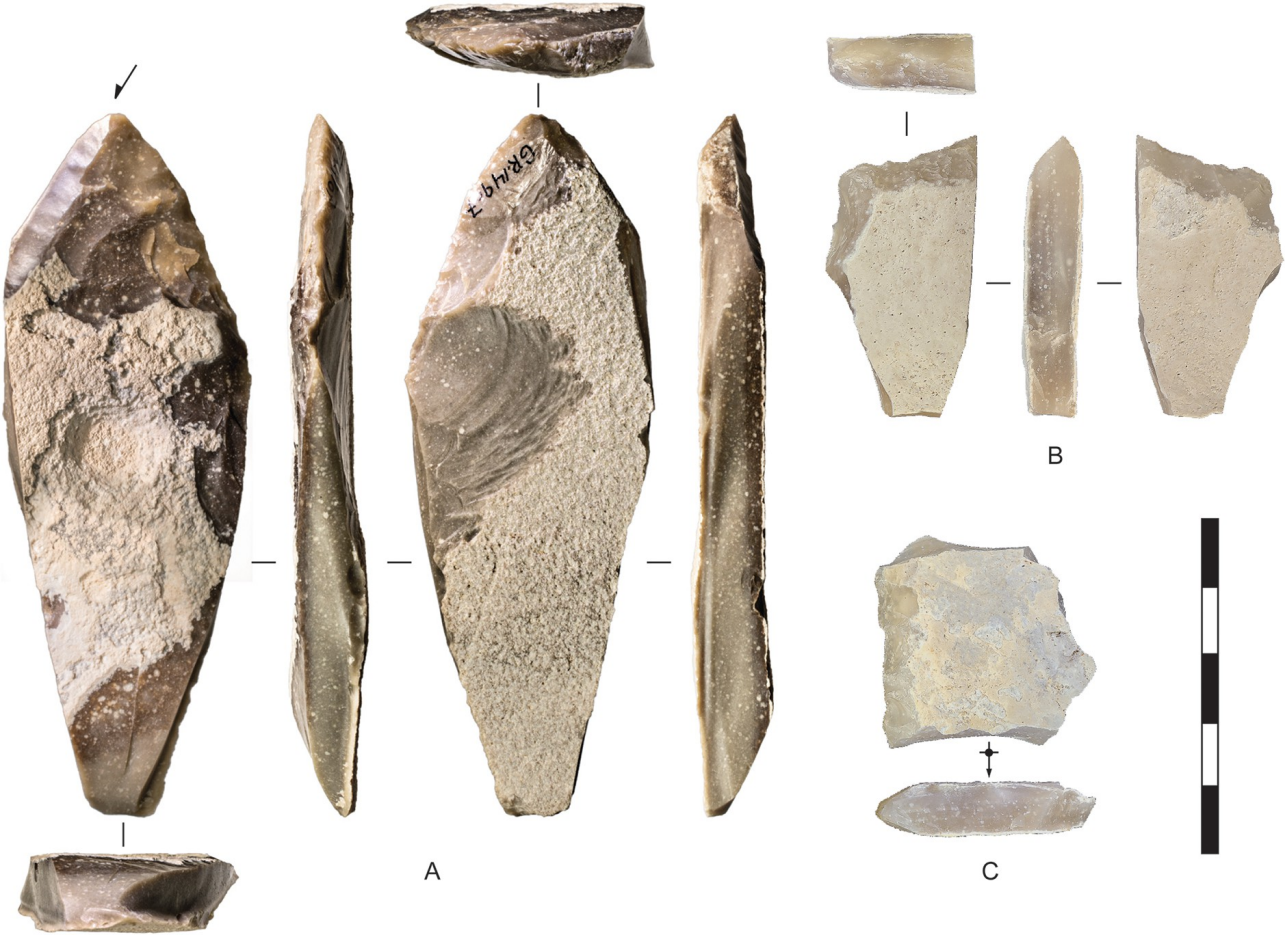
Bifacially-flaked chert and chalcedony tablets, Horizon IV, Saruq al-Hadid. (A) Chert tablet reduced by relatively invasive percussion flaking (images courtesy of Hélène David-Cuny). A burin-like blow was struck down one lateral edge from the tip, indicated by the arrow. This artefact was recovered from context 1793, but a wedge-shaped fragment with similar bifacial flaking-–probably from early in the same reduction event—was recovered from context 1790. (B) Chalcedony tablet reduced by steep, non-invasive bifacial percussion flaking, from context 1228. (C) Chalcedony tablet reduced by non-invasive bifacial percussion flaking, followed by sectioning on an anvil, from context 2448. The arrow points to a demicone on one of the truncated faces. Scale bar 50 mm.

The bifacial centripetal cores in the assemblage do not show evidence for anvil support. On these cores, flakes were removed from two faces, and were struck from a relatively low-angled bifacial platform. This produced relatively thick cores when applied to chert cobbles (Fig 16). Chert tablets were also reduced bifacially. Bifacial flaking on tablets was usually non-invasive, creating bifacial edges with relatively steep edge-angles, but in one case flakes were struck so they propagated a substantial distance across the face of the tablet, creating a low-angled edge (Fig 17). The chalcedony examples were truncated into segments, as discussed below.

Chert/chalcedony flakes struck at the site are relatively small, measuring on average 20.2 mm long, 4.5 mm thick, and weighing 2.2 grams (Table 4). Blows were struck an average of 2.9 mm from the core edge. Flake lengths and widths are highly variable, flake weight is exceptionally variable, and the coefficients of variation (CoVs) demonstrate a lack of standardisation in flake morphology. Platforms were never ground and rarely facetted (Table 3). Axial terminations were recorded on 16 flakes in contexts 1309 (N = 9) and 2008 (N = 7), showing that an attempt was sometimes made to strike flakes the entire length of the core face. Judging from the frequency of full-length flakes on core scars (see Figs 12 and 13), the low representation of axial terminations in the flake assemblage may indicate that axially-terminated flakes were disproportionately selected for backing.

**Table 4:** Dimensions of chert and chalcedony flakes, contexts 1309 and 2009, Horizon IV, Saruq al-Hadid.

	Length (mm) ^1^	Width (mm) ^1^	Length/Width^2^	Thickness (mm) ^1^	Platform depth (mm) ^1^	Weight (g)^2^
Number	252	326	232	366	243	231
Average	20.19	16.53	1.22	4.50	2.92	2.23
Maximum	51.94	46.77	3.43	24.52	12.61	28.28
Minimum	5.13	4.59	0.43	0.70	0.31	0.03
Standard Deviation	9.97	7.29	3.43	24.52	12.61	28.28
Coefficient of Variation (%)	49.40	44.08	39.39	64.76	72.08	144.69

^1^ Intact dimensions only.

^2^ Complete flakes only.

**Fig 18:**
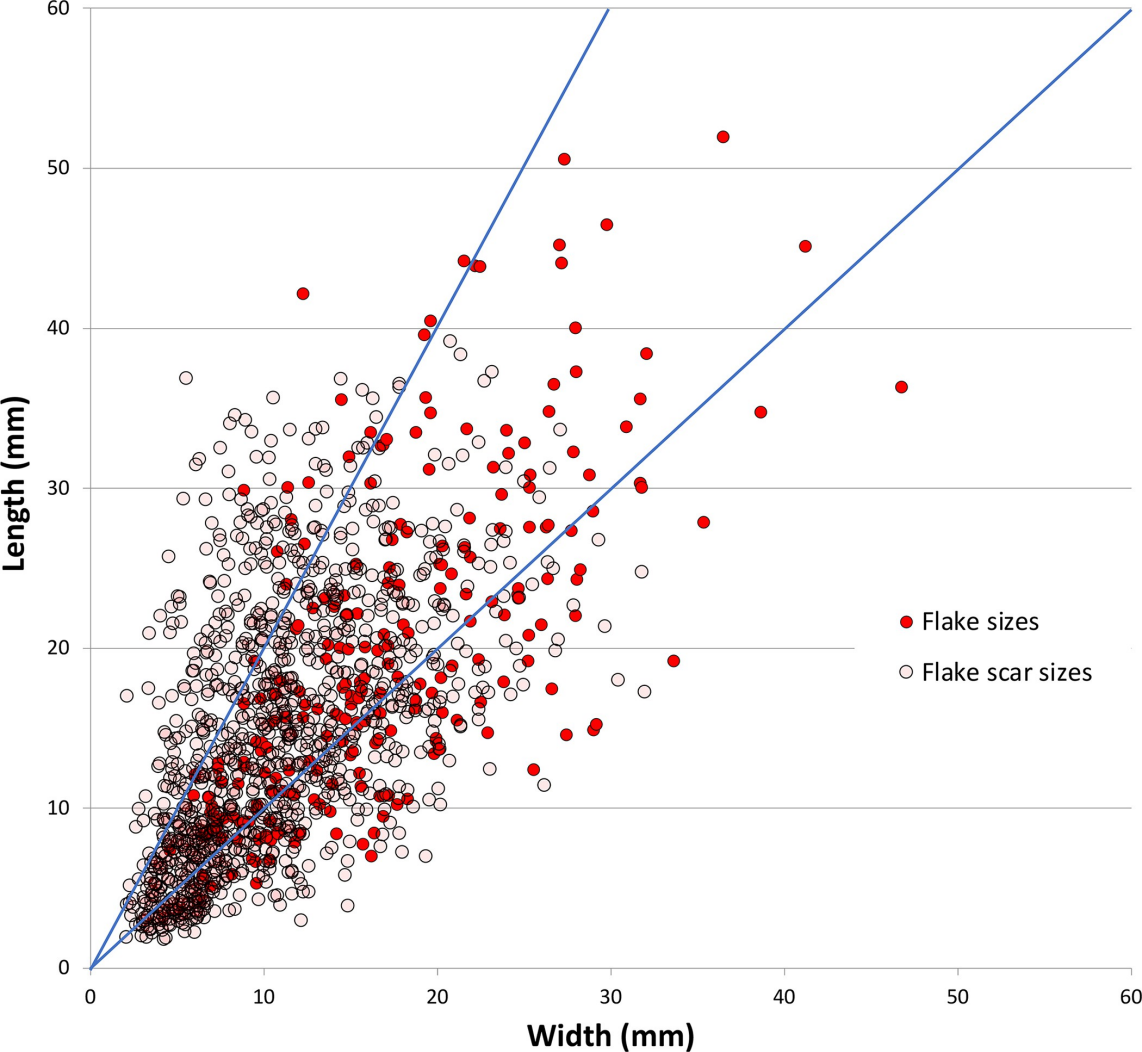
Scatterplot comparing the elongation of complete flakes and flake scars on cores, Horizon IV, Saruq al-Hadid.

Although the dorsal arrises on most flakes are parallel or sub-parallel, the flakes are only slightly elongated (length/width [L/W]); this variable is also relatively unstandardized (Table 4). Flakes are less elongated (L/W 1.22 ± 0.48, N = 232) than the scars on cores (L/W 1.49 ± 0.86, N = 1125). Although this difference is highly significant (p<0.0001, t = 4.6345, df = 1355, SE = 0.058), the degree of the difference in linear dimensions is relatively small (Fig 18). While some core scars and flakes are blade-like in morphology, on balance, Horizon IV stone-flaking is not a ‘blade’ technology in the conventional sense.

Flakes were recovered that are larger than the scars on discarded cores (Fig 18), in keeping with the reduction strategy of core rotation as reduction progressed, and progressive core attrition. The larger flakes were likely struck early in the reduction process. The similarity in flake and scar morphology supports an interpretation that most flakes were produced from cores reduced at the site, rather than imported from cores reduced elsewhere. Although uniface retouching occurred in Horizon IV, as indicated by the presence of unifacially-retouched flakes, no uniface retouching flakes were identified in contexts 1309 or 2008. Manufacture and resharpening of unifacial tools was uncommon relative to core reduction activities.

### Truncation and backing techniques

Freehand core reduction was sometimes done using anvil support, with blows oriented oblique to the core mass, as described above. A different anvil-support technique involved striking straight-in to the core mass. When the blow was applied near the centre of the blank’s surface, the object was broken in half or into sections of irregular shape. This variant of the technique is indicated by the presence of a demicone visible on the fractured edge of some artefacts.

‘Truncated pieces’ are the sections with negative truncation scar attributes, and ‘truncation segments’ are the sections with positive truncation scar attributes (a ventral surface), such as a positive demicone. If the blow was delivered close to the edge, the crack would initiate down the blank’s face, creating ‘planar backing flakes’. ‘Perverse backing flakes’ resulted from a spiral fracture mechanically identical to a perverse fracture in biface manufacture [after 60, 61]: rather than propagating down the backed face, as was likely intended, the flake instead propagated from the point of force application (PFA) at an angle to the backed face, breaking the segment into two pieces. The piece with the ventral surface is the perverse backing flake, and the piece with the negative scar is classified as a truncated piece if the section is irregular, or a microlith broken in manufacture if the section removed an unmodified chord opposite the backed face. Backed microlith production is described in more detail below.

**Fig 19:**
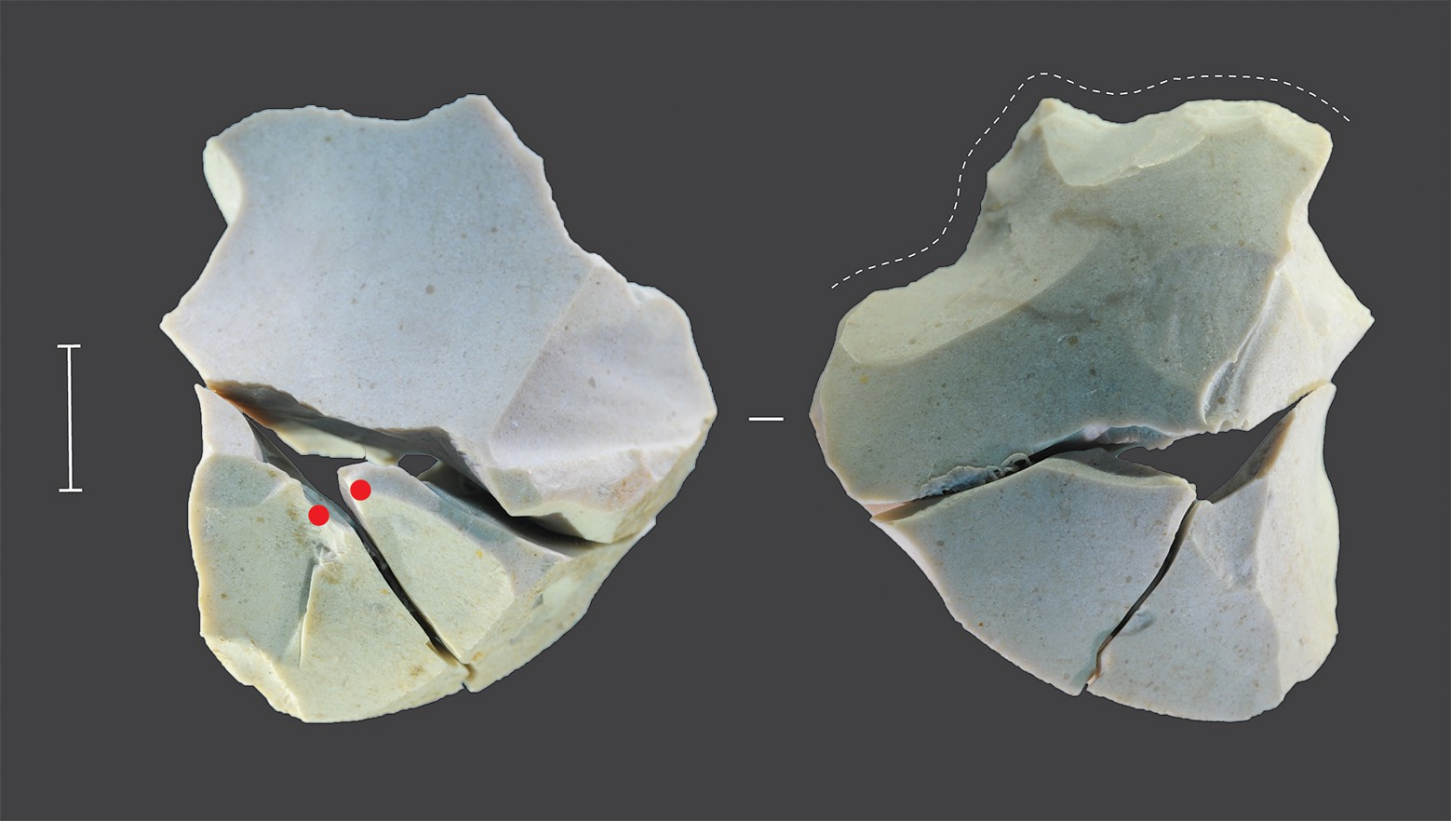
Conjoined set of truncated pieces, Horizon IV, Saruq al-Hadid. The ventral surface of the flake blank is shown on the right. Reduction began with unifacial freehand retouching to the blank’s ventral surface (indicated by the dotted line), and part of the edge shows microflaking, probably from use. This was followed by a series of anvil-supported truncations blows to the dorsal surface, culminating in the detachment of two conjoining truncation flakes. The points of force application for these two blows are shown by red dots. From context 2022, single reduction event (SRE) 32. Scale bar 10 mm.

Strong blows were delivered in truncating relatively thick blanks to ensure that the force was sufficient to ensure flake propagation through to the anvil support. Ventral attributes on the truncation byproducts tend to be distorted, with prominent compression ripples, and platforms are sometimes sheared or shattered. One flake truncated in this way was partially reconstructed through conjoining (Fig 19). Anvil support was also used in the backing variant of the technique, but in backing the blows were to the edge of the platform and were very carefully controlled.

**Fig 20:**
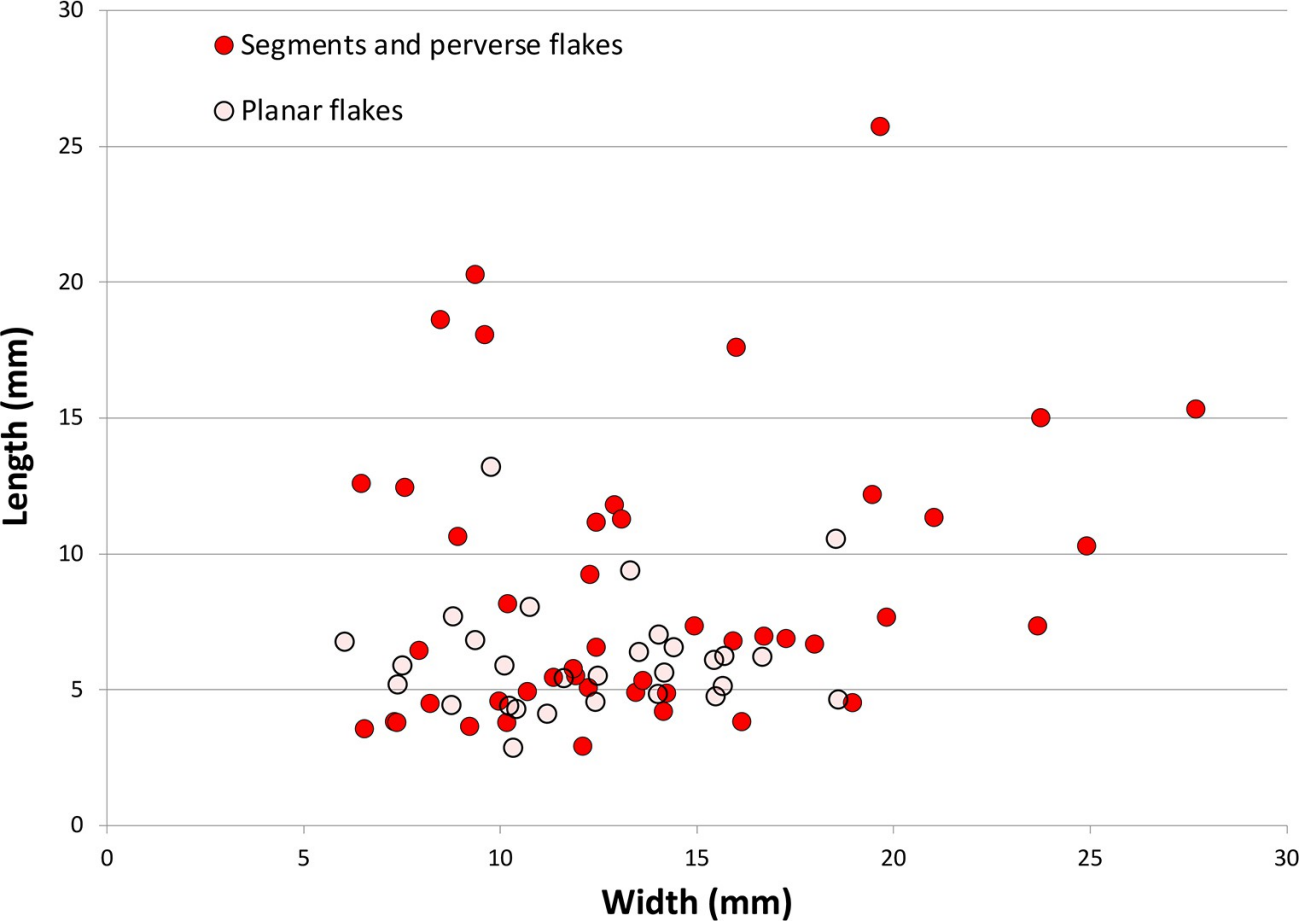
Scatterplot comparing the elongation of byproducts from truncating and backing flakes, Horizon IV, Saruq al-Hadid.

Truncation and microlith backing are ends of a technical continuum of flaking using anvil support, and the truncating/backing byproducts overlap morphologically. These byproducts tend to be wider than long (Fig 20) because their short length causes the propagation force to spread laterally across the backed face. As a methodological issue, it is not possible to clearly differentiate debris produced in the early stages of microlith production from debris produced in flake truncation; although microlith backing byproducts are at the smaller end of the size continuum, they overlap with truncation byproducts.

**Fig 21:**
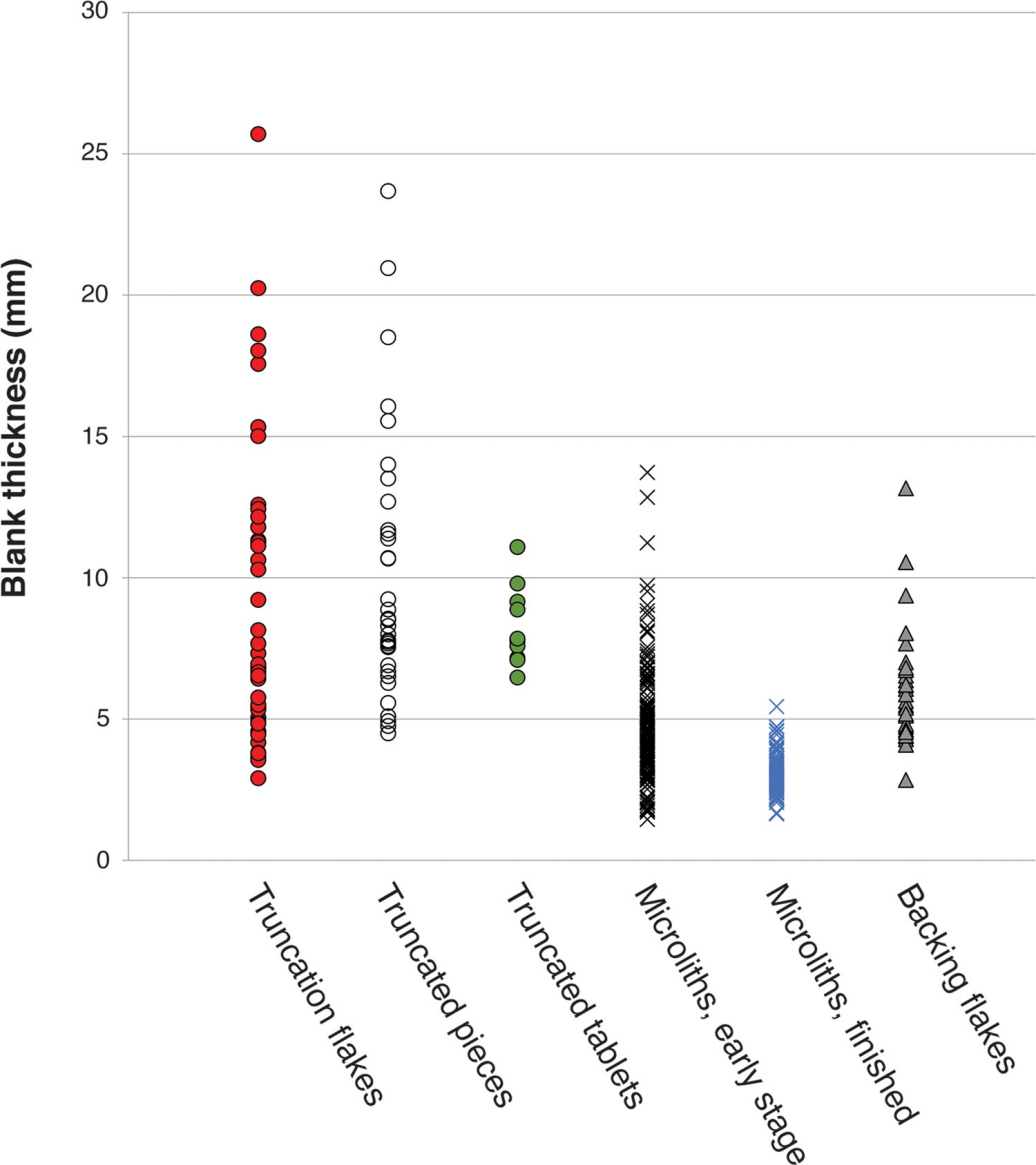
Thicknesses of blanks selected for truncating and backing, Horizon IV, Saruq al-Hadid. Thickness was measured directly on truncated pieces, microliths, and tile knives. Flake length is the proxy of blank thickness for truncation flakes and backing flakes.

**Fig 22:**
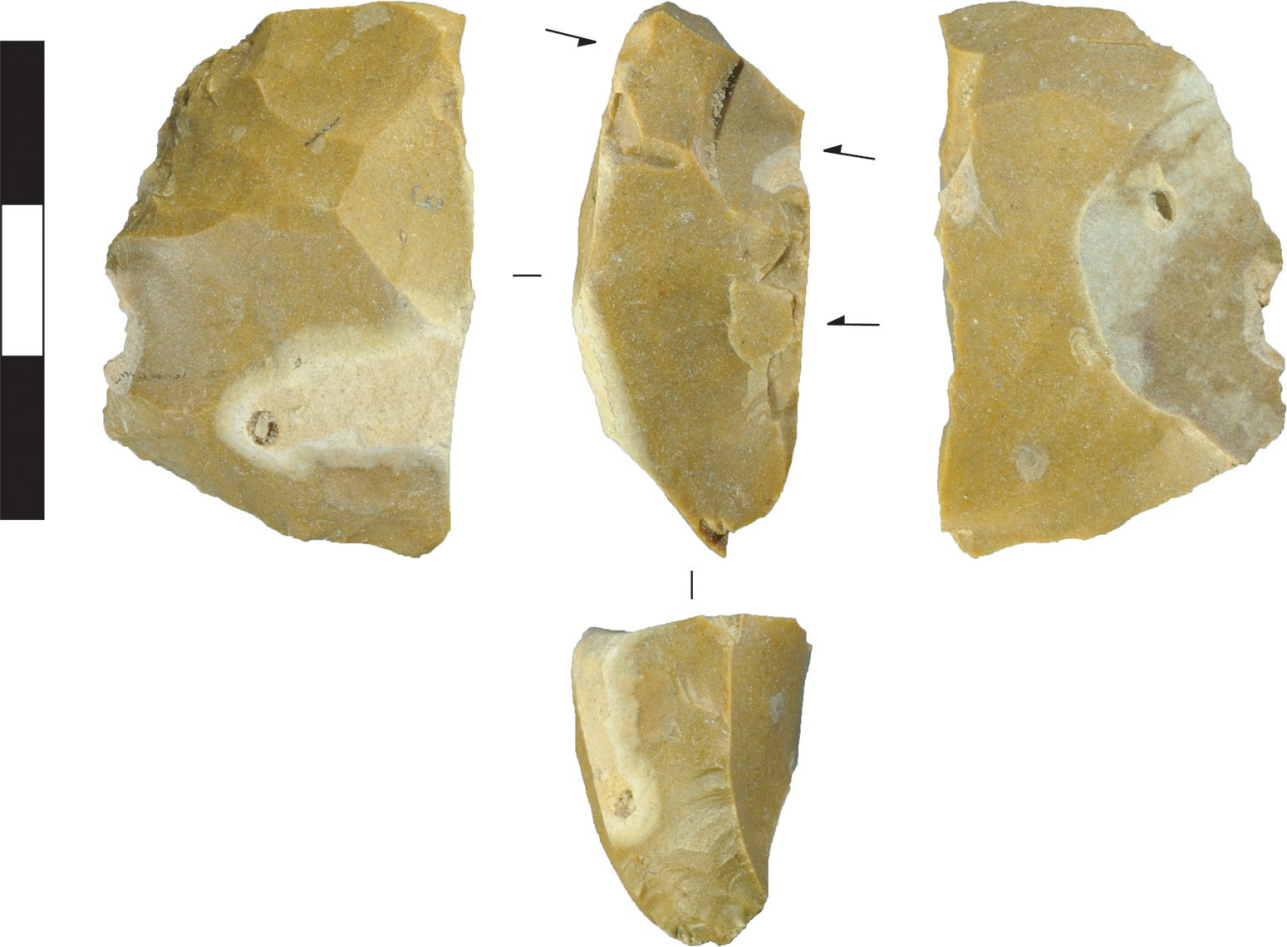
Chert bifacial centripetal core, Horizon IV, Saruq al-Hadid. The core was truncated by anvil-supported percussion (indicated by arrows). From context 2021. Scale bar 30 mm.

The truncating/backing process was one of shaping a retouched edge by attrition, much like slicing a loaf of bread, and in the same way that the length of a bread slice indicates the height of the bread loaf, the length of a truncation/backing flake roughly indicates the thickness of the blank (within a margin of variation due to varying thickness across the flake blank). Therefore the thickness of truncation pieces and backed microliths reflects the thicknesses of blanks selected for backing. A comparison of these values suggests that small, thin flakes—less than 10 mm thick, and mostly less than 5 mm thick—were selected for manufacture through backing into microliths, whereas thicker blanks—but usually less than 25 mm thick—were truncated (Fig 21). The larger flakes selected for truncating were likely from the early phases of core reduction, when the cores and flakes struck from them were still relatively large. The average thickness of the truncated flakes (9.99 ± 4.6 mm, N = 36) is over twice as large as the average thicknesses of the combined flake assemblage (4.49 ± 2.91 mm, N = 366, Table 9)—and the difference is statistically significant (p<0.0001, t = 10.2022, df = 400, SE = 0.539)—although they have a similar range of sizes. The truncation technique may have been employed to produce steep, durable edges on thick tools in some cases. This is most clearly suggested by truncated chalcedony tablet knives (Fig 17), which were first bifacially retouched and then truncated in a separate trajectory from backed artefacts. A bifacial centripetal core from context 2021 was also truncated (Fig 22), as were five recycled flakes from contexts 1748 (N = 2), 1794 (N = 1) and 2112 (N = 2).

### Microlith manufacture

**Fig 23:**
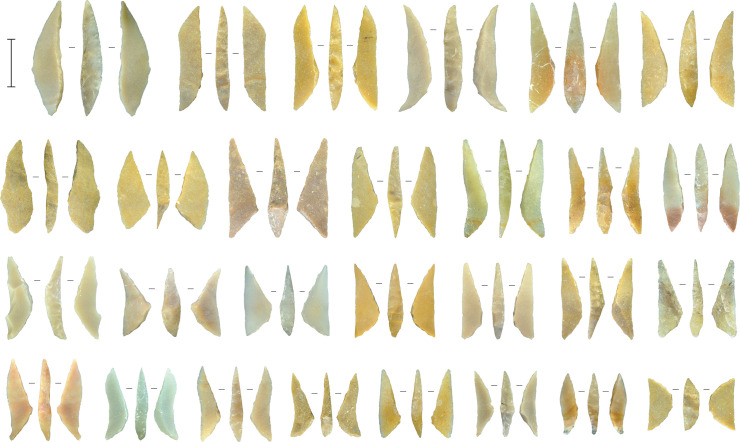
Backed microliths from Horizon IV, Saruq al-Hadid. Scale bar 10 mm.

**Fig 24:**
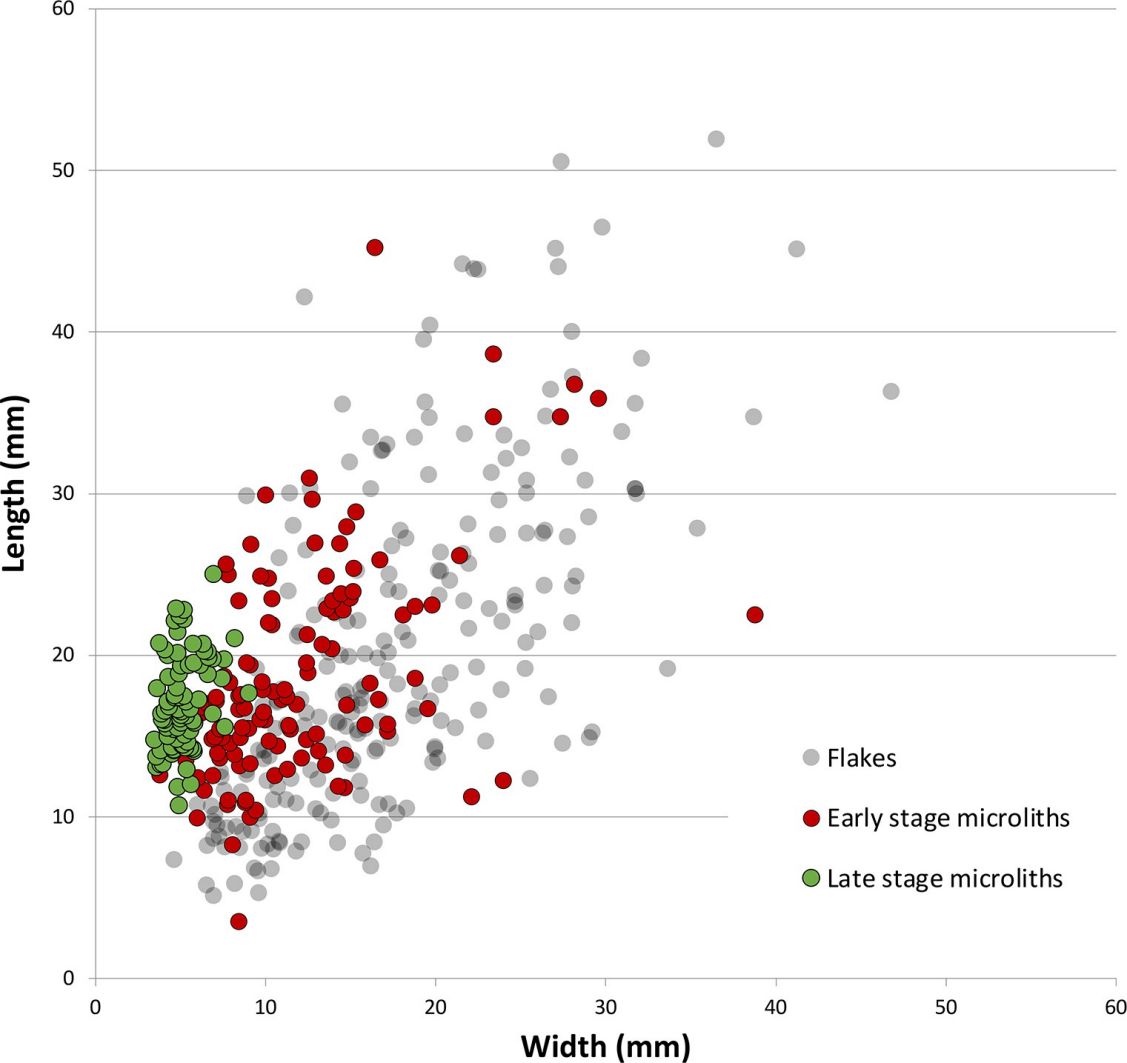
Scatterplot comparing the elongation of unmodified flakes and microliths from the early and late stages of backing, Horizon IV, Saruq al-Hadid. The backing process imposed size and shape constraints on the finished tools, and the data reflects the design criteria of the flintknappers.

The previous discussion demonstrates that the core reduction techniques practiced in Horizon IV at Saruq al-Hadid created morphologically diverse hard-hammer percussion flakes. This contrasts with the morphological similarity of the backed microliths (Fig 23). One focus of our analysis was to determine how the knappers produced relatively uniform microliths from varied flake sizes and shapes (Fig 24).

**Fig 25:**
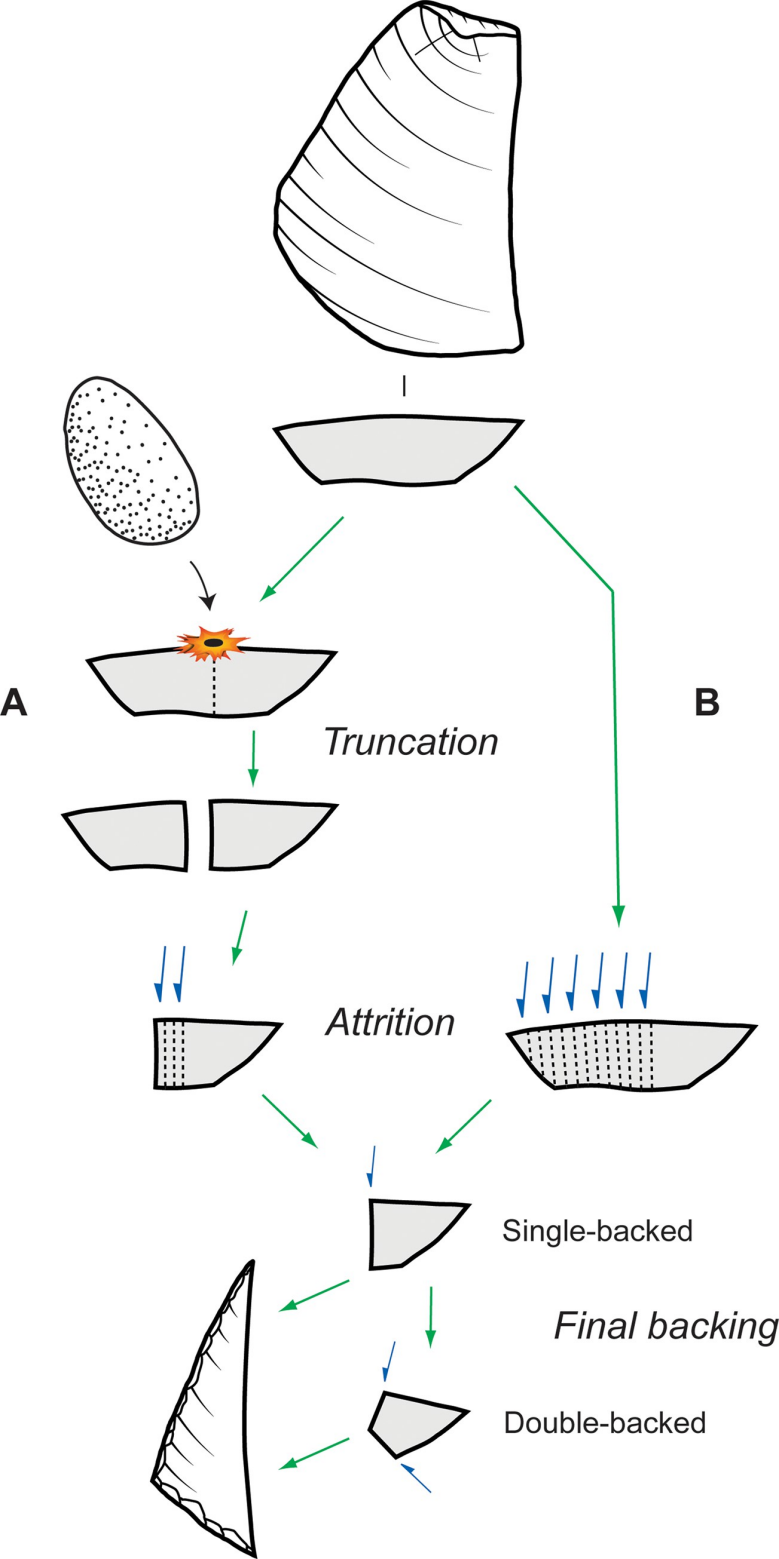
Schematic model of microlith backing methods, Horizon IV, Saruq al-Hadid. (A) Saruq Strategy A. (B) Saruq Strategy B.

Several SREs included debris from freehand core reduction, flake truncating, and microlith backing, showing that these techniques were practiced in the same reduction event. In these cases, both thick and thin blanks were struck from a core; sections of a thicker truncated flake (truncated pieces or segments) were progressively backing-retouched towards an intact former edge of the blank, which then became the chord of the finished microlith. This is referred to as ‘Saruq Strategy A’ (Fig 25). As attrition moved closer to the chord, the thickness of the backed object decreased. This is reflected in backing flake lengths, early stage backed microlith thicknesses, and finished microlith thicknesses (see Fig 21). Considerable attrition was necessary to produce microliths in this manner. Some 21% of the early stage microliths likely derived from Saruq Strategy A.

The more common strategy of microlith manufacture—‘Saruq Strategy B’—involved choosing flake blanks measuring less than about 8 mm thick (see Fig 21), thus avoiding a truncation stage. Some 79% of the early stage microliths were made in this way. As with Saruq Strategy A, the flake was initially narrowed through aggressive backing-retouch attrition until it was close to the final width.

**Fig 26:**
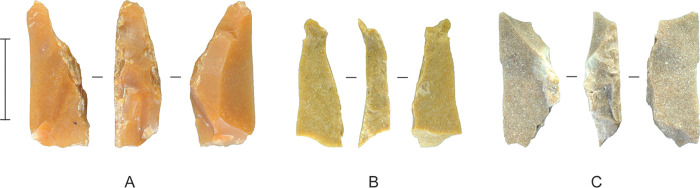
Microliths abandoned during the early phase of microlith manufacture, Horizon IV, Saruq al-Hadid. (A) Thick chert microlith with crushed platform edges and step-terminated flake scars on the backed face. The proximal end was detached by a single blow. (B) Chert microlith broken transversely across the proximal end, probably by a backing blow delivered without proper support. (C) Chert microlith with the distal end removed by a backing blow which propagated invasively and expanded laterally, rather than terminating at the anvil support. The lateral expansion lopped off the end of the microlith. From contexts 2023 (A), 2054 (B), and 2018 (C). Scale bar 10 mm.

Scars created during truncation/attrition are relatively large and irregularly spaced compared to the final backing scars, with more frequent platform crushing. About 77% of the microliths abandoned in manufacture were discarded during the early (truncating/attrition) or middle (attrition) stages of production (Fig 26; Table 5), attesting to the riskiness of the relatively heavy percussion technique used during attrition.

**Table 5:** Microlith stages, Horizon IV, Saruq al-Hadid.

Microlith stage	Context			Total
	1309	2008	Other	
Early (truncating/attrition)	4	5	42	51
Middle (attrition)	10	2	54	66
Late (final shaping)	1	1	34	36
Finished Microlith	1	6	87	94
Total	16	14	217	247

The orientation of the microlith within the flake blank was determined by assessing the direction of propagation from features on the ventral surface. Almost all microliths were oriented parallel or oblique to the direction of flake blank propagation, with the blank’s lateral margin forming the chord (Table 6). The distal edge of the flake blank was used as the chord in six cases.

**Table 6:** Backed microlith orientation relative to percussion axis, Horizon IV, Saruq al-Hadid.

Orientation	Reduction stage		Total
	Manufacturing rejects	Finished Microliths	
Oblique	1	2	3
Parallel	131	91	222
Right angle	5	1	6
Undetermined	16	--	16
Total	153	94	247

Most backing debris identified in the assemblage derived from the truncating/attrition stages of manufacture, including planar and perverse flakes. Planar backing flakes usually ended in an axial termination, are usually wider than long (Fig 20), and have ‘edge’ platforms from application of force on the platform margin. Planar backing flakes measure an average 6.1 ± 2.1 mm long (N = 29), but finished microliths measure an average of 3.03 ± 0.68 mm thick (N = 82). Given that backing flake length is a proxy measure of the thickness of the blank from which it was struck, most late-stage backing flakes were smaller than 3 mm in maximum dimension (see Fig 20) and were lost through the sieves.

**Fig 27:**
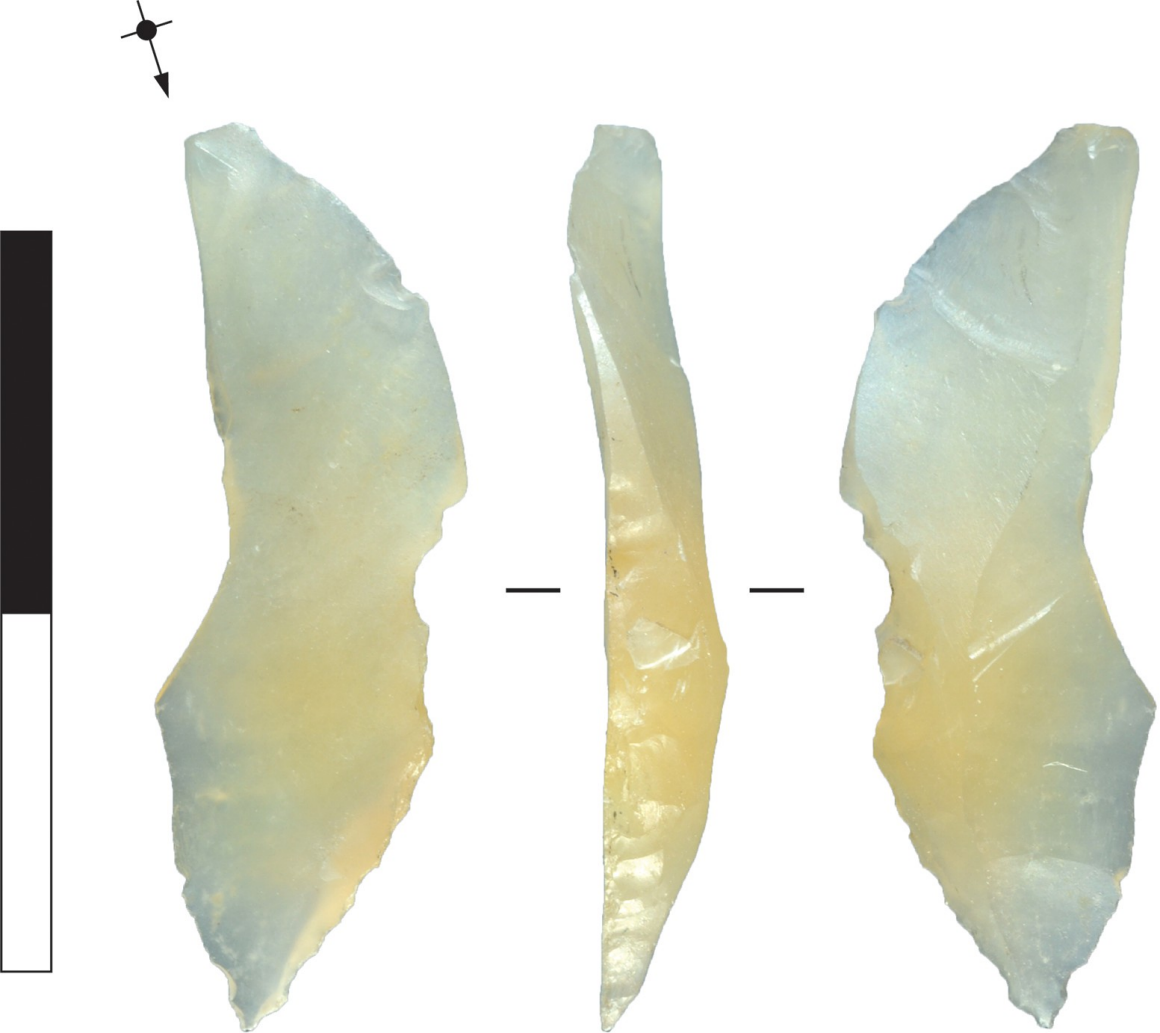
Chalcedony microlith abandoned in manufacture, Horizon IV, Saruq al-Hadid. The flake blank’s platform is intact and the proximal part of the flake is unmodified. The middle part of the microlith is coarsely backed, resulting in notching of the platform edge. The distal end of the microlith shows well-controlled, final-stage backing. From context 2040. Scale bar 20 mm.

**Fig 28:**
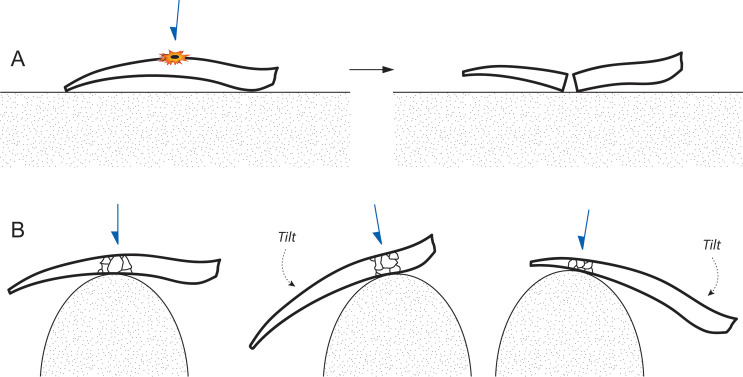
Technical issues in microlith backing, Horizon IV, Saruq al-Hadid. The schematic drawing shows the side view of a flake blank and anvil surface. (A) If a flake blank is placed on a flat anvil, there is a risk that the blank’s curvature will cause it to be unsupported opposite the point of force application (PFA), and backing can fail through a bending fracture. (B) Effective backing occurs when the blank is properly supported opposite the PFA. A convex surface allows the flintknapper to maintain anvil contact regardless of the degree of flake blank curvature, by tilting the blank as backing progresses.

The outline of microliths abandoned during the attrition stage tend to be irregular, with PFAs spaced apart from each other along the backed edge. The platform margin is in some cases somewhat ‘notched’ from using a relatively narrow-ended indentor and delivering the blow back slightly from the platform edge (Fig 27). As with flake truncating, the middle and late stages of microlith manufacture also appear to have relied on anvil support. In a few cases, the line of propagation between the platform and termination on planar backing flakes suggests that the anvil surface was excurvate rather than flat. When backing is applied to a blank placed on a flat anvil, it can be turned, but tilting the blank upwards is not possible because that gesture lifts most of the stone from anvil contact. With a curved anvil, the blank can be both turned and tilted, maintaining contact with the anvil at all times (Fig 28). This allows backing to be applied more effectively to blanks that are curved or irregular in long section. Backing attrition, then, was likely accomplished with a narrow indentor and a curved anvil, such as a cobble edge.

**Fig 29:**
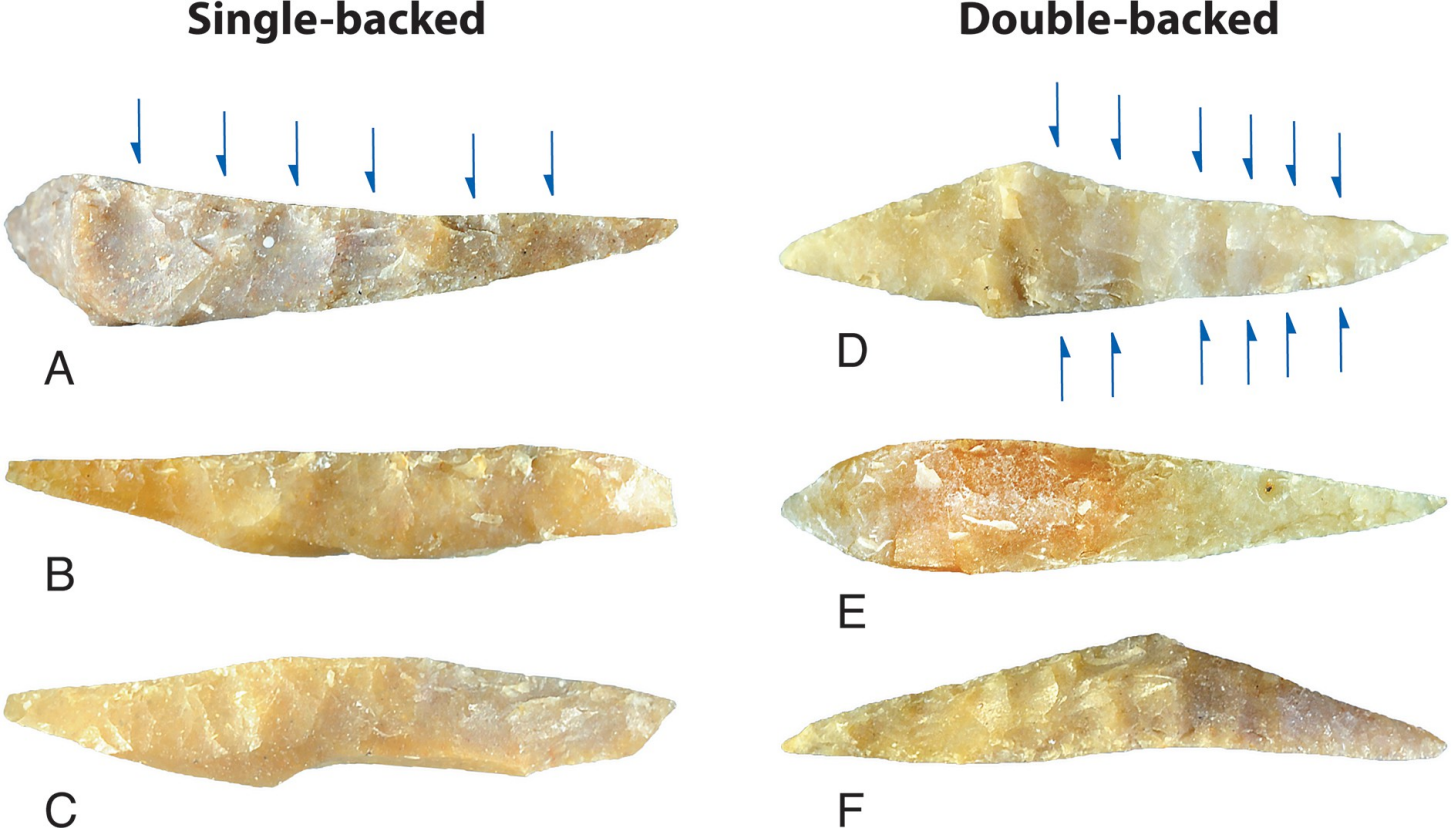
Single- and double-backed microliths, Horizon IV, Saruq al-Hadid. In the top examples, the arrows indicate the directions of the main flake scars. From contexts 2022 (A), 2008 (B), 2321 (C), 2049 (D), 2822 (E), and 1748 (F). Not to scale.

Backing flakes were initiated from one platform edge, creating single-backed microliths, or directly opposed platform edges, creating double-backed microliths (Fig 29). Single-backing was sometimes applied to opposite platforms in different areas of a backed microlith, referred to here as ‘single x 2’ backing. Early stage microliths—broken and rejected in manufacture—are more likely to be single-backed, but finished microliths are often double-backed (Table 7). This suggests a shift in technique from early/middle stage attrition to final stage finishing. Backing scars from the earlier stages of manufacture are more likely to be preserved on the ‘short leg’ of finished asymmetrical microliths; final-stage finishing was used to produce the delicate ‘long leg’ and, in many cases, a needle-sharp distal end (see Fig 23).

**Table 7:** Single- and double-backed edges relative to microlith reduction stages, Horizon IV, Saruq al-Hadid.

Backing	Reduction stage		Total
	Manufacturing rejects	Finished Microliths	
Single	82	11	93
Single x 2	17	6	23
Double	49	76	125
Unclassified	5	1	6
Total	153	94	247

**Fig 30:**
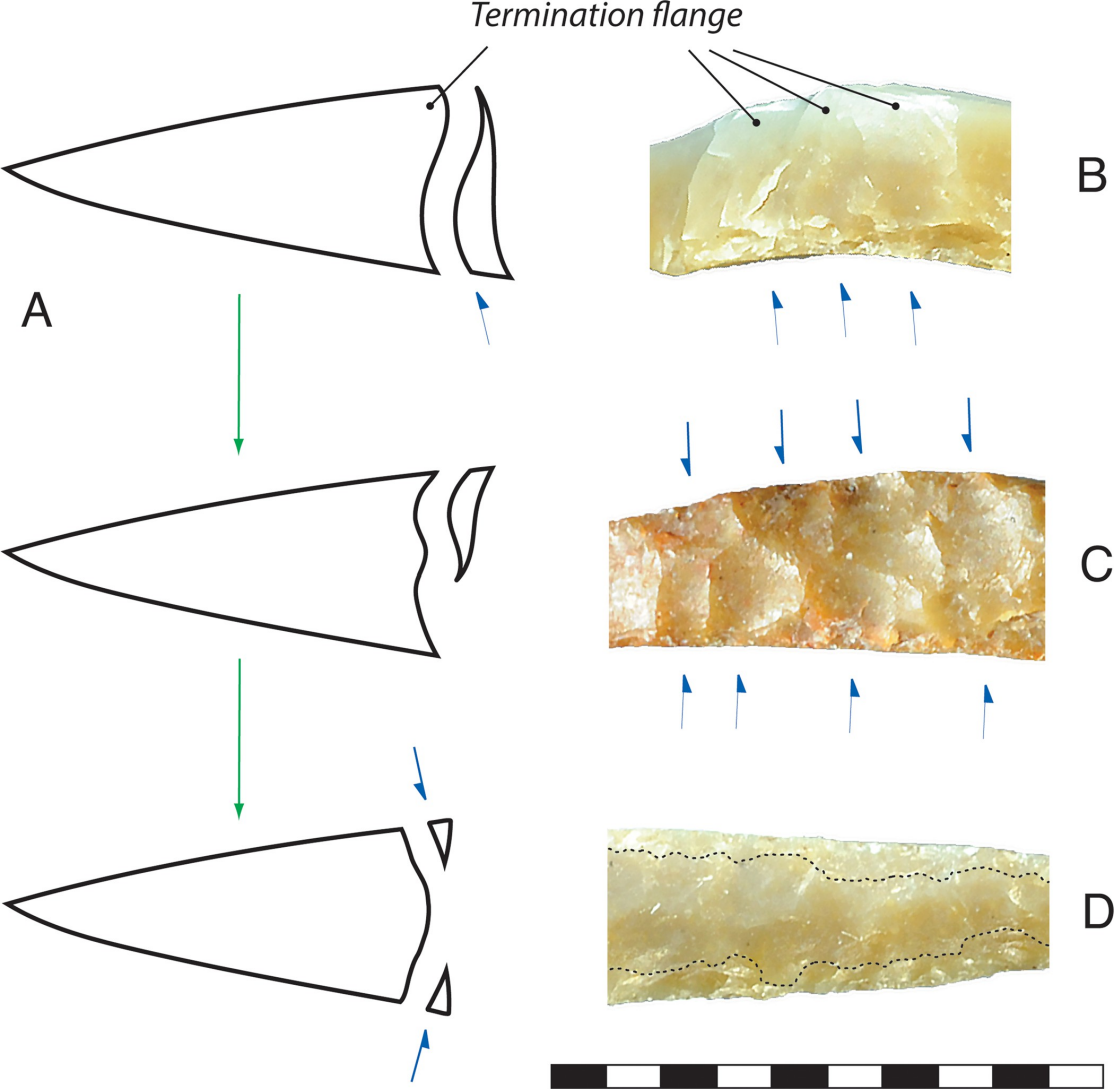
Late stage microlith backing method, Horizon IV, Saruq al-Hadid. (A) Schematic cross-section view of late stage microlith backing sequence. (A, top) Full-length backing flakes can create scars with a zone of high mass near the termination end, distal to the relatively deep negative bulb of percussion. This mass is referred to as the ‘termination flange’. (A, middle) The termination flange is removed by flakes struck from the opposite edge of the backed face, terminating near the middle. (D, bottom) The ‘overhang’ proximal to the percussion bulbs are removed by a non-invasive raking technique, enhancing the domed profile to the backed face. (B) Examples of termination flanges on 3 flake scars. The arrows indicate the directions of the principal flake removals. (C) Examples of flange removal scars, indicated by the top arrows, terminating near the middle of the backed face. (D) Example of a domed face enhanced by a raking technique, creating non-invasive scars. The dotted lines show the approximate limit of the raking scars. Scale bar 10 mm, for artefact D. From contexts 1794 (B, D) and 2009 (C).

In final stage finishing, the microlith was carefully manipulated and the points of applied force were spaced closer together than during the attrition stage. The delicacy of many finished microliths may indicate that final-stage backing was accomplished by anvil-supported pressure-flaking, rather than percussion (see [62]). On many microliths, final-stage double-backing was achieved in three steps (Fig 30). In Step 1, flakes propagated completely across the backed edge, resulting in an axial termination on the backing flake. This created a relatively flat profile to the backed surface and backing sometimes ended at this point, or skipped to Step 3. However, negative bulbs were often pronounced, and the reorientation of the crack from the bulb to the termination resulted in a recurved surface called a ‘termination flange’. In Step 2, the microlith was flipped and the flange was eliminated by removing flakes that terminated near the middle of the backed face. In Step 3, the sharp overhanging edges from steps 1 or 2 were removed by edge-raking, detaching microflakes and creating relatively non-invasive flake scars. The result of this two- or three-step process was a slightly ‘domed’ backed surface profile. The prominence of the dome depended on the depth of the bulbs from steps 1 or 2 combined with the amount of attrition caused by edge raking. Final-stage finishing appears to have proceeded from the distal end of the flake blank towards the proximal end, and a ‘domed’ morphology is often only present on the long leg of the microlith; the short leg of a microlith is more likely to display scars from Step 1 backing.

Raw material colours of finished microliths are broadly similar to other artefact types, including early stage microliths, although a greater proportion of finished microliths are made from ‘other’ chert/chalcedony materials (Table F in S1 Table). Conversely, finished microliths of orange/yellow materials are relatively rare. The similarities in raw material colours indicate that most late-stage microliths were manufactured at Saruq al-Hadid, but the differences may suggest that some microliths were manufactured elsewhere and carried to the site as finished tools.

### Microlith morphology

**Fig 31:**
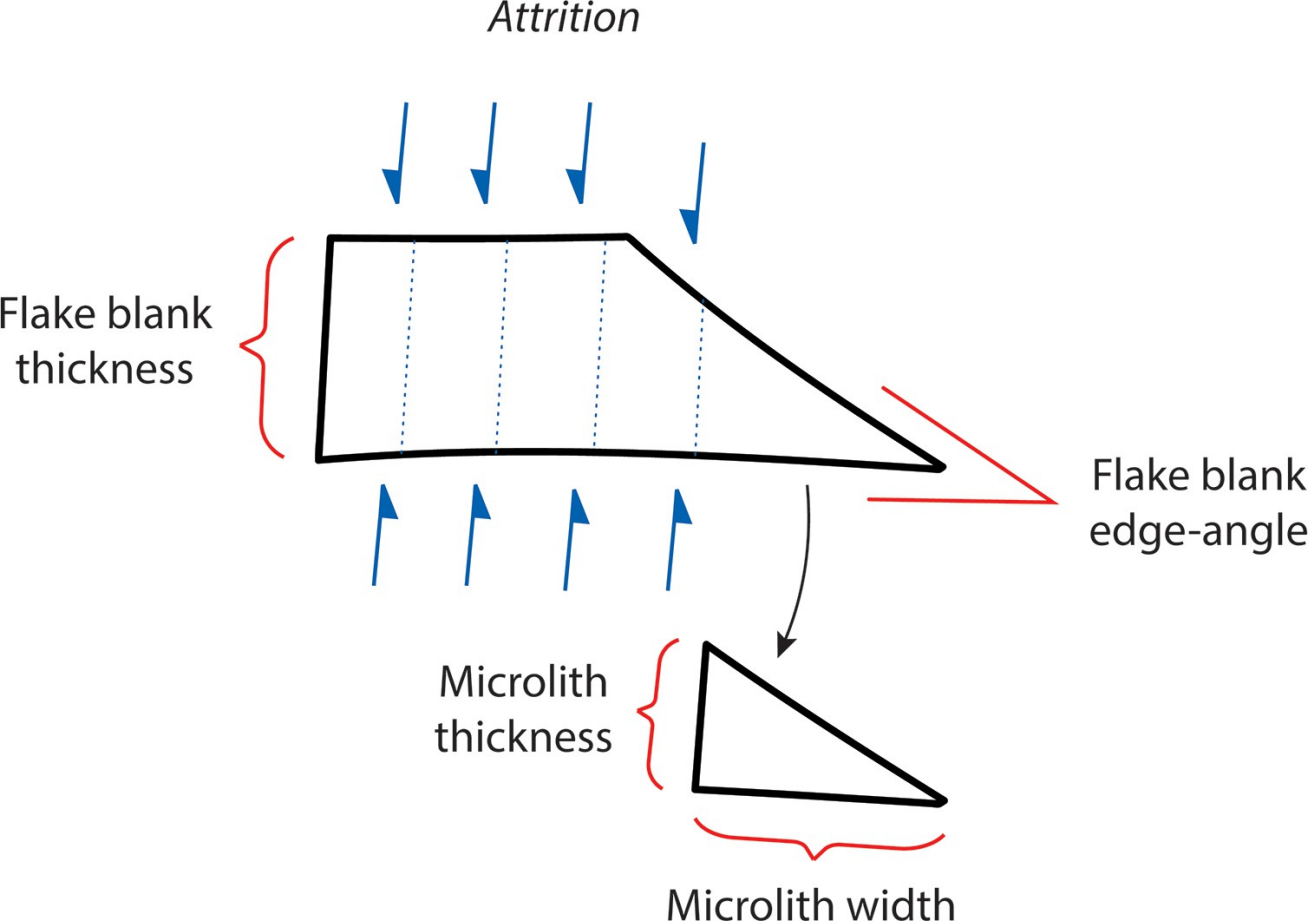
Schematic cross-section of flake attrition and backing, Horizon IV, Saruq al-Hadid. The drawing illustrates how the thickness of a finished microlith may not reflect the thickness of the flake blank. Attrition progresses until the microlith falls within the desired thickness range; the width of the finished microlith will be strongly influenced by the edge-angle of the flake blank.

In Saruq al-Hadid microlith manufacture, the thickness of the final microlith is contingent on backing attrition relative to the edge-angle of the flake combined with the desired microlith width, not the maximum thickness of the flake as detached from the core (Fig 31; Tables 8 and 9). By significantly decreasing the width of the blank through attrition (Saruq Strategy A), combined with careful selection of smaller blanks (Saruq Strategy B), relatively standardised thicknesses (CoV 21.82%) could be achieved on unstandardized flake blanks (CoV 64.76%). Similarly, the elongation ratio of completed microliths is high (3.41 ± 0.76) relative to unmodified flake blanks (1.22 ± 0.48), an effect achieved through attrition in backing rather than from the blank production strategy. Significant shaping through attrition is technically risky in the early stages, as attested by the relatively large number of backing failures recovered in the assemblage. It is also an inefficient use of stone, as the flakes may be considerably larger than the microliths made on them.

**Table 8:** Dimensions of chert and chalcedony microlith manufacturing rejects, Horizon IV, Saruq al-Hadid.

	Length (mm) ^1^	Width (mm) ^1^	Length/Width^2^	Thickness (mm) ^1^	Platform depth (mm) ^1^	Weight (g)
Number	116	151	116	153	28	153
Average	18.99	11.65	1.69	4.74	3.81	1.21
Maximum	45.22	38.79	3.33	13.73	8.64	16.58
Minimum	3.53	3.80	0.42	1.45	1.27	0.1
Coefficient of Variation (%)	36.41	45.72	35.68	43.46	45.63	168.99

^1^ Intact dimensions only.

^2^ Complete artefacts only.

**Table 9:** Dimensions of chert and chalcedony finished microliths, Horizon IV, Saruq al-Hadid.

	Length (mm)*	Width (mm)*	Length/Width**	Thickness (mm)*	Tip Cross Sectional Area ^1^ (TCSA), square mm		Maximum Width Position ^2^ (MWP)	Backed Artefact Symmetry Index ^3^ (BASI)	Weight (g)
					Lengthwise	Crosswise			
Number	82	94	82	94	94	82	46	46	94
Average	17.08	5.32	3.41	3.02	8.16	26.19	33.69%	0.67	0.22
Maximum	25.03	9.42	5.59	4.74	21.28	57.69	49.64%	0.99	0.64
Minimum	10.75	3.45	1.97	1.65	3.33	11.31	16.65%	0.33	0.08
Standard Deviation	2.94	1.23	0.76	0.66	3.10	8.56	7.81%	0.16	0.10
Coefficient of Variation	17.20%	23.15%	22.15%	21.82%	38.04%	32.68%	23.19%	23.19%	44.39%

* Intact dimensions only.

** Complete artefacts only.

^1^ See [63]. For microliths mounted lengthwise, the TCSA is calculated as (0.5 x W) x Th. For microliths mounted crosswise, the TCSA is calculated as (0.5 x L) x Th (after [64]: 2600).

^2^ After [65]: 13. MWP is calculated as (L_M_/L_T_) x 100, where L_m_ is the shortest length from an end of a microlith to the position of maximum width, and L_T_ is the maximum length. The largest possible value is 50% for a perfectly symmetrical microlith.

^3^ After [66]. The BASI index calculates the largest possible MWP as 1.0 (MWP 100%) rather than 0.5 (MWP 50%), for a perfectly symmetrical microlith. By convention, the BASI index is not converted to a percent.

Late-stage microliths varied least in length and varied most in width, as measured by CoV values, although metrical variation was greatest for the linear measurements of length, and variation was least for thickness and width (see Table 9). Despite these statistical variations, absolute size tolerances were within about 2 mm for width and thickness, 6 mm for length, and 0.2 grams. These measures of variation are much greater for the early stage microliths (see Table 8), and the decrease in metrical variation from the early to the late stage (Fig 24) reflects the design intentions of the Saruq al-Hadid flintknappers. On balance, it would appear that they were most concerned with the width and thickness of the microliths, with length more likely to vary; weight correlated with these other size values. This likely reflects the hafting arrangement. For instance, if inset into a groove or notch in the side or end of an organic shaft, width and thickness are key variables: microlith thickness is important to the width of the notch or groove, and width is important to the depth of the notch or groove. Variation in length is less important.

**Fig 32:**
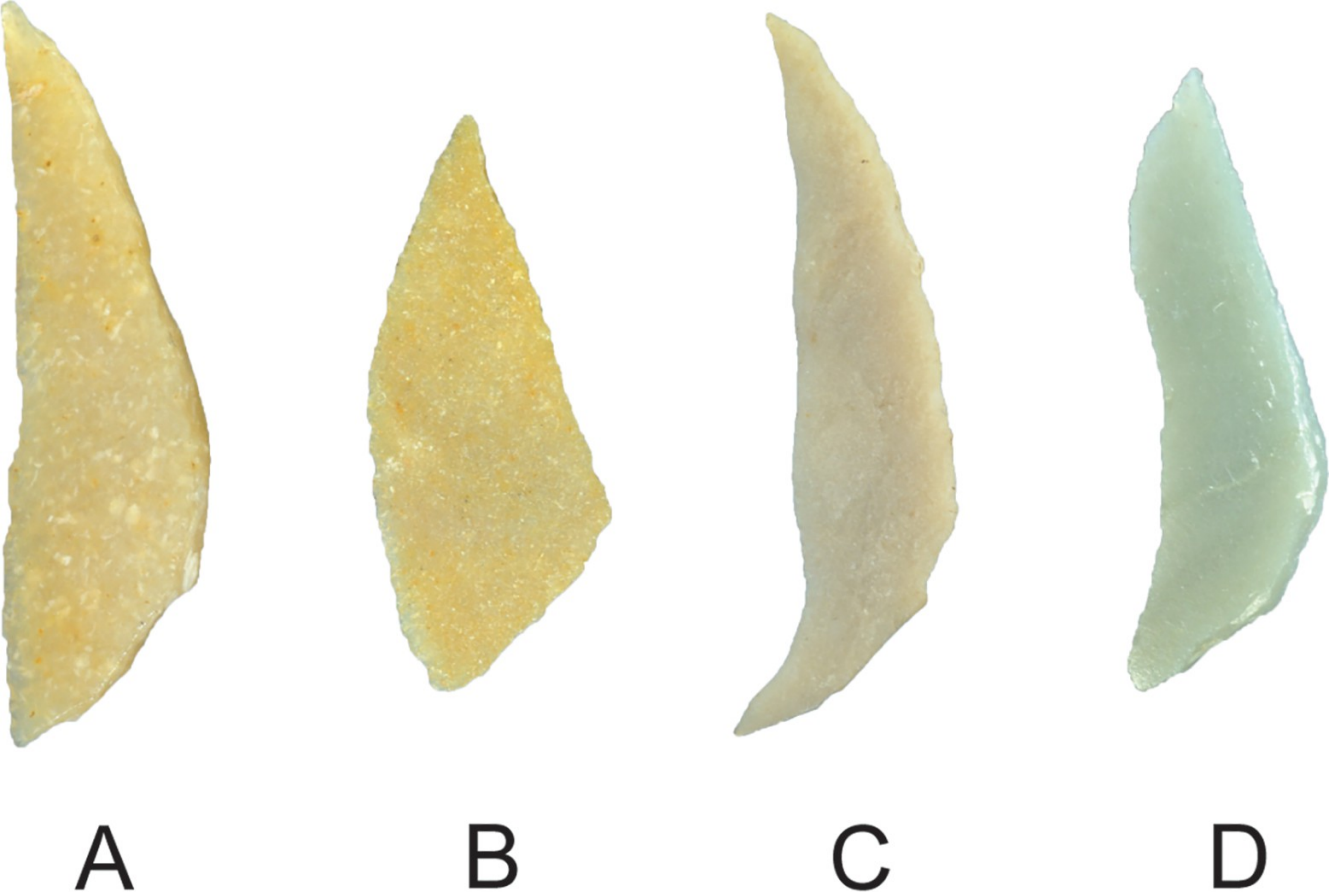
Examples of microlith chord shapes, Horizon IV, Saruq al-Hadid. (A) Straight chord, context 2026. (B) Excurvate chord, context 2232. (C) Incurvate chord, context 2008. (D) Sinuous chord, context 1794. Not to scale.

A minority (38%) of the late-stage Saruq al-Hadid microliths had straight chords; most (62%) curved inward, outward, or were a sinuous mixture of the two (Fig 32, Table 10). This reflects the unstandardized approach to flake production, which was not overly concerned with the shape of the flake blank edge. It also indicates that edge straightness was not a primary consideration for the intended purpose.

**Table 10:** Backed microlith chord shapes, Horizon IV, Saruq al-Hadid.

Chord shape	Reduction stage		Total
	Manufacturing rejects	Finished Microliths	
Excurvate	40	25	65
Incurvate	24	9	33
Straight	55	31	86
Sinuous	13	29	42
Not classified	21	--	21
Total	153	94	247

**Fig 33:**
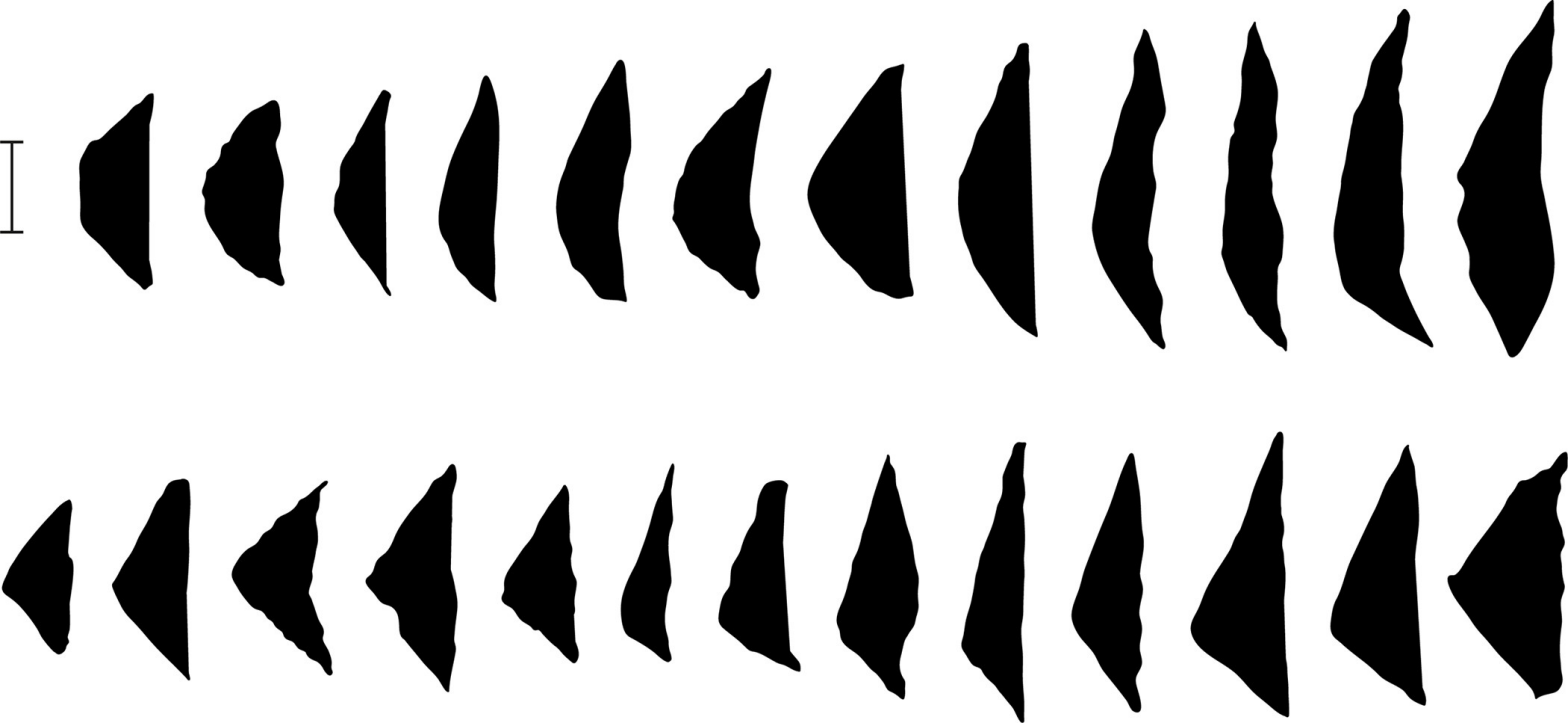
Microlith silhouettes showing the continuous variation in shape Horizon IV, Saruq al-Hadid. Scale bar 10 mm.

The shapes of the backed edges of early-stage microliths were irregular, reflecting the less-controlled percussion technique used in Step 1 attrition. In contrast, many of the late-stage microliths were expertly-backed and show a consistency in shape that suggests ‘style’. Some are symmetrical, with a half-moon shape, but a more common stylistic variant is classified as ‘scalene’. Scalene microliths are asymmetrical, with the shorter backed leg meeting the longer backed leg at an angle of about 110–130 degrees. The angle is abrupt on many examples, but this shape grades into artefacts with a similar asymmetry but a less-accentuated angle (Fig 33).

**Fig 34:**
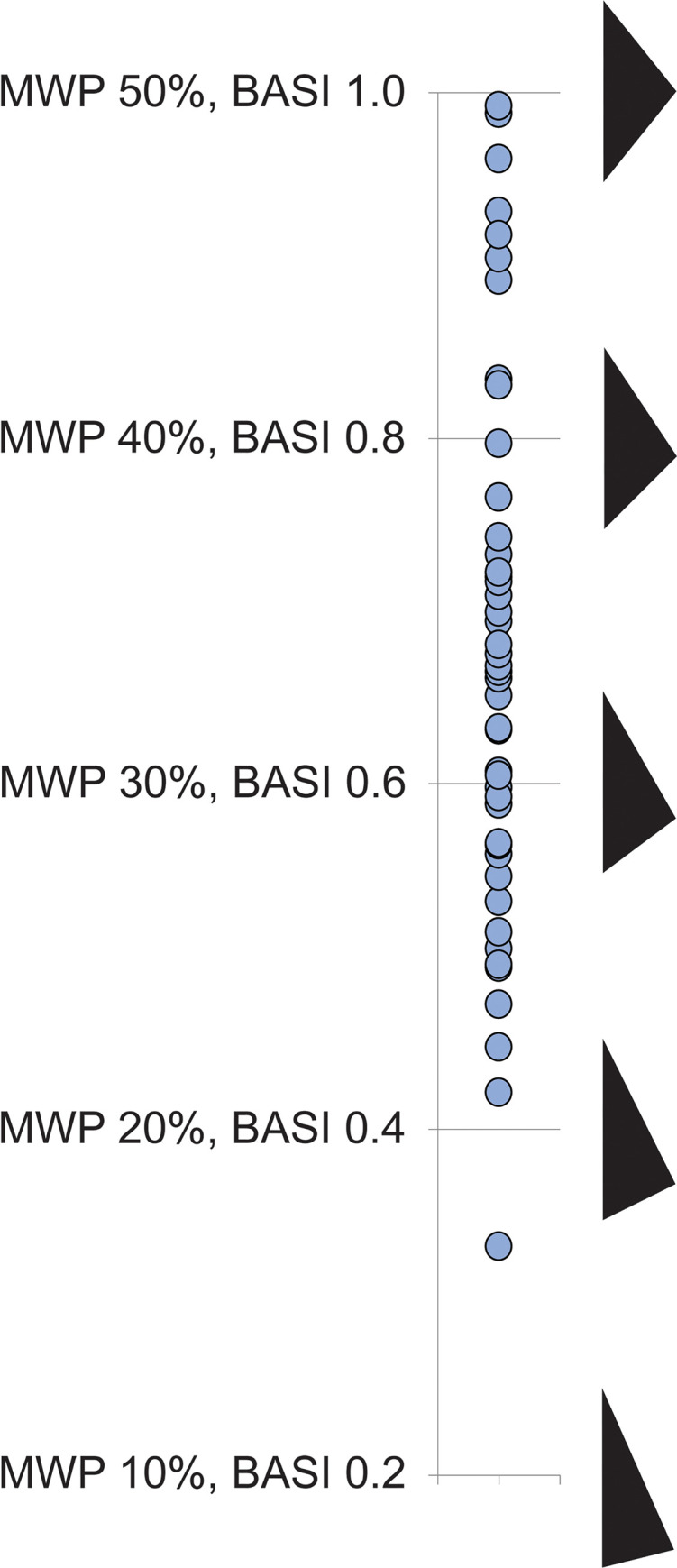
Symmetry data for finished microliths from Horizon IV, Saruq al-Hadid. Perfect symmetry is 50% in Maximum Width Position (MWP) [65] and 1.0 in the Backed Artefact Symmetry Index [66]. The proportions are illustrated with schematic silhouettes of scalene microliths.

Asymmetry can be assessed empirically by calculating the Maximum Width Position (MWP), or the location of the widest point of the microlith as a proportion of the total length (after [65]). On a perfectly symmetrical microlith, the MWP is 50%: the widest point is at the centre of the blank. On average, the short leg on a final stage Saruq al-Hadid microlith is about one-third (33.7%) of the microlith’s total length (see Table 9, Fig 34). About 13% of the finished microliths are highly symmetrical (MWP >45%). A modified version of MWP, called the Backed Artefact Symmetry Index (BASI) [66] assigns the value 1 to the 50% MWP and calculates a value between 0 and 1 for the proportional distance of an artefact’s maximum width to the MWP; a BASI index of 1 indicates perfect symmetry. Finished Saruq al-Hadid microliths have an average BASI index of 0.67 ± 0.16 (Table 9). Regression analysis indicates that MWP asymmetry in Saruq al-Hadid microliths is poorly correlated with weight (R^2^ = 0.00073) or chord length (R^2^ = 0.01993), suggesting that microliths were shaped to similar proportions regardless of blank size.

Variation in the ‘tip cross sectional area’ (TCSA) may distinguish between the stone armatures of different types of hunting/fighting weapons [63]. TCSA is a proxy of the penetration ability of the point. As such, the TCSA measurement is designed to distinguish weapon tips; backed microliths may have been hafted lengthwise as weapon tips, but archaeological and ethnographic evidence suggests that they may have been hafted as side elements (rendering TCSA irrelevant), or crosswise (with the chord oriented at right angles to the shaft) [35]. TCSA measurements for the finished microliths at Saruq al-Hadid (Table 9) fall within Shea’s [63] arrowpoint values for both lengthwise and crosswise configurations (after [64]). Although the TCSA metric seems robust for ethnographic arrowpoints, it poorly distinguishes dart points, with some ethnographic dart point armatures falling within Shea’s arrowpoint values [35, 67].

The metrical variation at Saruq al-Hadid can be contrasted with Australian microliths because both technologies involved the production of flake blanks using hard-hammer percussion. The Australian method was similar to Saruq Strategy B (pers obs. MWM) and involved less invasive attrition during blank shaping than that seen in Saruq Strategy A. Australian microliths are metrically more similar to the early-stage manufacturing rejects from Saruq al-Hadid than the final microliths (Table 11), suggesting the Saruq al-Hadid knappers were working within tighter design parameters. This may be a reflection of different use: Australian microliths were used for both spear armatures [68] and cutting implements [69], but not arrowpoints. Also, Australian hafting resins are exceptionally strong [70] and it is unlikely that Australian microliths were affixed into a groove; hence, design tolerances for microlith thickness, in particular, may have been looser in Australia.

**Table 11:** Dimensions of backed microliths from southeast Queensland, Australia, and Horizon IV, Saruq al-Hadid.

Provenance	Calculation	Length (mm) ^1^	Width (mm) ^1^	Length/Width^2^	Thickness (mm) ^1^	Weight (g)
Southeast Queensland microliths, finished	Number	106	156	106	158	95
	Average	21.84	10.48	2.27	4.97	1.93
	Standard Deviation	8.42	4.79	1.05	3.98	4.20
	Coefficient of Variation (%)	38.54	45.67	46.26	80.14	217.24
Horizon IV microliths, manufacturing rejects	Number	116	151	116	153	153
	Average	18.99	11.65	1.69	4.74	1.21
	Standard Deviation	6.88	5.33	0.60	2.06	2.04
	Coefficient of Variation (%)	36.41	45.72	35.68	43.46	168.99
Horizon IV microliths, finished	Number	82	94	82	94	94
	Average	17.08	5.32	3.41	3.02	0.22
	Standard Deviation	2.94	1.23	0.76	0.66	0.10
	Coefficient of Variation (%)	17.20	23.15	22.15	21.82	44.39

^1^ Intact dimensions only.

^2^ Complete artefacts only.

## Discussion

Although a blade-based technology with relatively large backed microliths dates from 30,000–33,000 years ago in Oman [71], this technology does not extend continuously into later periods. Backed microliths are very rare in early to mid-Holocene Neolithic assemblages in southern Arabia, which tend to be dominated by projectile points made on large blades, or, later in the sequence, pressure-flaked bifaces.

The expression of stone-flaking technology varies markedly at southeastern Arabian sites of the subsequent periods. For instance, backed microliths are absent and lithic artefacts and production debris are rare on many sedentary settlement sites of the Bronze and Iron Ages. Large-scale excavations produced very small lithic assemblages at sites such as Tell Abraq ([72]: Table 4; [73]: 50), Bat [74], Kalba K4 and Shimal SX (D. Eddisford, pers. comm. to LW; C. Velde, pers. comm. to LW). However, the recent excavations at Hili 8 suggest that these low numbers are at least in part a reflection of recovery methods, as high-retention collection strategies utilizing sieving have generated lithic assemblages an order of magnitude larger than those from earlier work at the site ([75]: 139). Larger flaked stone assemblages—technologically similar to the Saruq al-Hadid assemblage but lacking backed microliths—were recovered from Hafit and Umm an-Nar sites at Ra’s al-Hadd in Oman, and are interpreted as specialist toolkits for processing shell [76, 77].

Backed microliths are also absent from the lithic assemblages from nearby short-term Bronze Age occupation and hunting sites analogous to Saruq al-Hadid, such as Al-Ashoosh and Al Sufouh 2 [31, 78, 79]. Other artefact classes show comparable dissimilarities in contemporary Wadi Suq/Late Bronze Age assemblages, highlighting the difficulty of distinguishing between cultural and functional differences from archaeological patterning. Theoretically, Horizon IV at Saruq al-Hadid could reflect a distinct, mobile hunter-gatherer or pastoral community separate from, but economically integrated with, the sedentary communities across the region. Conversely, the stone assemblage at Saruq al-Hadid might reflect a task-oriented toolkit used by members of largely sedentary communities who undertook seasonal hunting activities away from their home base. Our current hypotheses regarding the Horizon IV occupation of Saruq al-Hadid contain some elements of both of these models; we identify a broad cultural unity in the region, but highlight the pervasiveness of individual and group mobility in the creation and maintenance of this ‘dispersed community’ [5].

Recently, however, evidence has emerged that the production of backed microliths at Saruq al-Hadid has local antecedents in the third millennium BC. Excavations at Hili 8 in the UAE have recovered 8 backed ‘lunate’ microliths. Possible technological links with earlier Neolithic stone technologies (see [35]) are tenuous, an assessment shared by Buchinger et al., who regard the microliths from Hili 8 as a new, locally-evolved technology ([75]: 137). More generally, backed microlith industries of comparable age to those from Horizon IV at Saruq al-Hadid are reported from southwestern Arabia, northeastern Africa, the Levant, Iran, and South Asia (see [35]). Backed microliths from the Tihamah coast in southwestern Arabia are made on obsidian from across the Red Sea in Africa, and are elements of an ‘Afro-Arabian cultural sphere’ ([80]: 91) that includes local microlith-producing traditions [81] (see also [82]: Fig 154). An Iron Age stone artefact assemblage from HAS1 (ca. 800 BC-200 AD), on the coastal plain of Salalah in southern Oman, is technologically similar to the Saruq al-Hadid assemblage, with least-effort chert core reduction to produce blanks for small backed microliths [83]. Stone tool assemblages from Bronze Age Harappan or Indus Valley traditions in Gujarat include backed microliths (e.g., [84]: 498–513, Fig 8.197), including scalene triangles similar to those found at Saruq al-Hadid ([85]: Fig 2). These and other examples suggest a very widespread microlith-using technological tradition during this period [35] and persisting into the Iron Age.

The function of backed microliths in these various lithic traditions remains a matter of debate. Reviewing the long-term use of obsidian geometric microliths in northeastern Africa and southern Arabia by societies with diverse subsistence practices, Khalidi et al. simply note that they were ‘[u]sed to make composite tools whose function remains unconfirmed’ ([80]: 81) The Early Bronze Age examples from Hili 8 are interpreted as inserts for sickles and linked to the introduction of agriculture in the region during the third millennium BCE. Specifically, Buchinger et al. claim: ‘[d]esign and development of this new type served one specific task that emerged with the introduction of agriculture: cutting large amounts of soft plant fibres, in other words, harvesting cereals’ ([75]: 144). The same function is proposed by Inizan and Francaviglia ([86]: 17), who regard first millennium BCE geometric obsidian microliths from Yemen as sickle elements, although noting that their use may have extended to the cutting of a variety of plants, not just cereals.

In contrast, a non-agricultural function is proposed for the Iron Age microliths from HAS1 in southern Oman [83], despite the presence at the site of ground-stone implements that may have been associated with plant processing. Noting the absence of sickle gloss on any of the lithics from the site, the authors suggest that they may have been used for a variety of tasks including ‘[n]et repairs, food processing, animal butchering, and other cutting activities’ ([83]: 190).

An agricultural use does not seem likely for the small backed microliths from Saruq al-Hadid, given the site’s desert environment and their occurrence in a midden dominated by the remains of hunted wild animals, in which remains of cereals were extremely scarce ([6]: Table 4). Furthermore, the absence of sickle gloss on any of the studied microliths from the site does not support their use for harvesting cereals or processing any other types of silica-rich plants.

**Fig 35:**
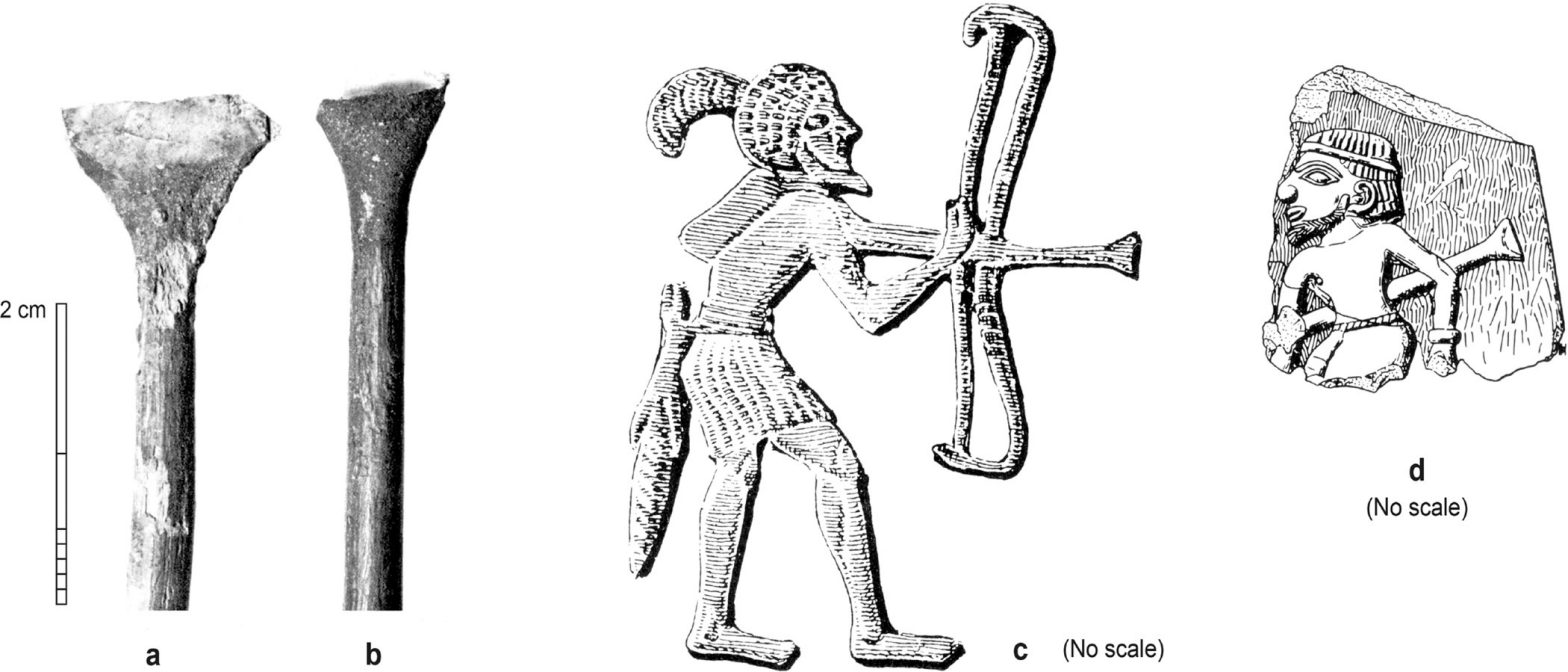
Microliths from late prehistory. (a, b) Hafted transverse backed microlith arrowheads from tombs at Naga-ed-Der, Egypt, ca. 2182–2052 BC (after [88]: Plate V). (c, d) Transverse arrowheads depicted on slate palettes from Hierakonpolis, Egypt (c), and lower Egypt (d), ca. 3650–3300 BC (after [88]: Fig 1). Figure by Hélène David-Cuny.

Elsewhere we have argued that the backed microliths at Saruq al-Hadid principally functioned as armatures for arrows [35]. This identification has previously been proffered for backed microliths from southern Arabia ([82]: 329) and is common for backed microliths in metal-age site assemblages in wider Southwest Asia [87]. Support for this hypothesis is provided by arrows with single hafted microliths of similar age documented from Egypt [88, 89] (Fig 35) and portrayed in 7^th^–8^th^ Century BCE reliefs from Yemen [90].

The backed microliths from Saruq al-Hadid span the mid-second millennium BC and thus occur alongside the first documented metal arrowheads in southeastern Arabia ([91]). If they can indeed be regarded as components of projectile points–a functional interpretation whose detail and proof awaits traceological analyses–they add significant nuance to our understanding of the reintroduction of archery to southern Arabia after a ‘dark millennium’ in which this technology is not attested by archaeological evidence from the region [91–93].

The Saruq al-Hadid lithic reduction technology reflects a relatively simple, least-effort approach to core reduction, but flake backing demonstrates a high degree of technical skill. This is also true of the Tihamah sites, where microliths were made on flakes from a least-effort bipolar core reduction technique [80, 81], and the somewhat earlier Early Bronze Age backed microliths in the Negev, which were made on ad hoc flakes or blade-like flakes [94]. The later Iron Age microliths from Oman were also made on ad hoc flakes ([83]: 188). Rosen [87] proposed that social disruptions at the beginning of the second millennium BC in the Levant caused the demise of specialised flintknappers who produced standardized stone blanks for sickles, and production shifted to ad hoc blank manufacture by the artisans who assembled backed stone elements into sickles. This shift provided the economic context for the transition to metal sickles. Moore et al. [35] propose that microlith technology at Saruq al-Hadid reflects a similar pattern, with wasteful, ad hoc core reduction providing blanks for microliths by the arrow-making artisans themselves. This pattern contrasts, however, with the Harappan backed microliths, which appear to have been made on pressure blades [85], requiring highly-skilled and specialist core reduction techniques [95].

## Conclusion

Excavations at Saruq al-Hadid have produced a large assemblage of securely provenanced stone tools from the Wadi Suq/Late Bronze Age, ca. 1750–1300 BCE. The stone tools were made and discarded in the context of a dense midden containing wild oryx, gazelle, and camel bone, as well as the remains of domesticated goat and sheep, fish, birds, and other small animals.

Chert for flaked-stone tools was procured as small cobbles from a variety of distant primary geological outcrops, and scavenged from nearby stone artefact scatters, and the materials were transported to the site after minimal assaying at the source. Transport costs were low because the stones were so small. At Saruq al-Hadid they were reduced into flakes through a simple freehand or anvil-supported hard-hammer percussion technique. Cores were rotated frequently as they were reduced and the reduction strategy was least-effort, done in response to the developing morphology of the cores rather than through a hierarchical series of flaking stages. Thin chert or chalcedony plates were retouched bifacially by hard-hammer percussion to create tablet knives. Some of the larger flakes, as well as the tablet knives, were truncated by striking the face in the middle, creating angular pieces with steep edges, perhaps for use as tools. Some flakes were unifacially retouched. The approach to this aspect of the reduction technology was ad hoc and relatively low-skilled, but higher skill levels are reflected in microlith manufacture. Flakes from ad hoc reduction were selected for the production of backed microliths. The early stages of microlith production often involved substantial attrition by anvil-supported hard-hammer percussion. Final stage backing was far more controlled and frequently involved a delicate double-backing technique, possibly accomplished using pressure, that often resulted in a distinctive ‘domed’ backed edge profile. The stoneworkers were more concerned with the widths and thicknesses of the microliths than the lengths, and the repetition of certain shapes—particularly scalene triangles—suggests attention to microlith ‘style’.

A picture is now emerging of a broadly-contemporary Bronze Age regional interaction sphere involving backed microliths, likely hafted as projectile points. Similar backed microliths were produced in a band from Africa and Egypt, through Arabia and the Levant, to Gujarat in India and Luristan in Iran. Although the technologies used to produce blanks for microliths included different forms of ad hoc knapping, such as the direct percussion method at Saruq al-Hadid and the bipolar method on the Tihamah coast, highly technical blank production methods were used at Harappan sites in India. The varying environmental and cultural contexts of this Bronze Age microlith technology—as well as the varied methods of producing them—may indicate that stone-tipped arrows played a role in social signalling [96, 97]. This is particularly relevant given the shift towards metal arrowheads during the Bronze Age, and it promises to provide fresh insights into the complexity of social changes occurring in Arabia and beyond during this critical period.

## Supporting information

S1 Table Context and stone artefact data, Horizon IV, Saruq al-Hadid. (DOCX)
