# Supplementary material for: Bronze age stone flaking at Saruq al-Hadid, Dubai, southeastern Arabia
Source: PLoS One. 2022 Jul 13;17(7):e0270513. doi: 10.1371/journal.pone.0270513 (PMC9278746; doi:10.1371/journal.pone.0270513)
Supplement: S1 Table — (DOCX) [file pone.0270513.s001.docx]

**Supporting Information**

**S1 Table: Context and stone artefact data, Horizon IV, Saruq al-Hadid.**

**Table A. Archaeological contexts subjected to full lithic analysis, Saruq al-Hadid,**

**Horizon IV.**

| **Context number** | **Description** | **Volume sieved (decalitres)** ^1^ | **No. of stone artefacts** | **Density**  **(stone artefacts per decalitre)** |
| --- | --- | --- | --- | --- |
| 1309 | Deflated deposit at the top of the bone layer (Horizon IV), ca. 50 mm thick, overlying sloping dune surface. Bone was densely packed with stone artefacts dispersed throughout. Bone density 15.4 kg per cubic metre. | 162.0 | 292 | 1.80 |
| 2008 | Intact context within the bone layer (Horizon IV) comprising a ca. 150 mm thick matrix of fine, pale brownish yellow sand with common ash lenses, bone, pottery sherds, and lithics. Bone density 4.9 kg per cubic metre. | 310.5 | 230 | 0.74 |

^1^ All of the deposits in these contexts were dry-sieved through 3 mm mesh.

**Table B. Colours of chert and chalcedony flaked-stone artefacts, Horizon IV, Saruq al-Hadid.**

| **Colour group** | **Munsell**^1^ | **Colour variety** | **Context 1309** | | | **Context 2008** | | | **Other contexts** | | | **Total,**  **N (%)** |
| --- | --- | --- | --- | --- | --- | --- | --- | --- | --- | --- | --- | --- |
|  |  |  | **Chal** | **Chert** | **N (%)** | **Chal** | **Chert** | **N (%)** | **Chal** | **Chert** | **N (%)** |  |
| Brown/Tan | 10YR 5/4 | Brown | 1 | 42 | 77  (29.3%) | -- | 32 | 79  (40.7%) | 5 | 82 | 330  (68.6%) | 486  (51.8%) |
|  | 10R 4/6 (stripes) | Brown and red striped | -- | 2 |  | -- | 14 |  | -- | 15 |  |  |
|  | 10YR 7/4 | Tan | -- | 20 |  | -- | 32 |  | 8 | 197 |  |  |
|  | 10R 4/6 (stripes) | Tan and red striped | -- | 12 |  | -- | 1 |  | -- | 23 |  |  |
| Orange/Yellow | 10YR 8/6 | Orange | 3 | 10 | 119  (45.3%) | 4 | 16 | 71  (36.6%) | -- | 3 | 16  (3.3%) | 206  (22.0%) |
|  | 5Y 8/4  5Y 7/6 | Yellow | 1 | 92 |  | 5 | 42 |  | 13 | -- |  |  |
|  | 10R 4/6 (rings) | Orange with red rings | -- | 13 |  | -- | 4 |  | -- | -- |  |  |
| White/gray | 5Y 7/2  5Y 8/1  10YR 8/2 | White/gray | 2 | 42 | 44  (16.7%) | 4 | 31 | 35  (18.0%) | 29 | 73 | 102  (21.3%) | 181  (19.3%) |
| Red | 5YR 3/4  10R 4/6  5YR 5/6 | Red | -- | 4 | 17  (6.5%) | 2 | 5 | 7  (3.6%) | 6 | 15 | 21  (4.4%) | 45  (4.8%) |
|  | 10YR 8/2 (spots) | Red with white spots | -- | 13 |  | -- | -- |  | -- | -- |  |  |
| Other | N3  5GY 3/2 | Black | -- | 2 | 3  (1.1%) | -- | -- | 2  (1.0%) | -- | -- | 8  (1.7%) | 13  (1.4%) |
|  | 5G7/4  5GY 6/1  5GY 7/4  10GY 6/4 | Green | -- | -- |  | -- | -- |  | 4 | -- |  |  |
|  | 5YR 8/4 | Pink | -- | 1 |  | -- | 2 |  | 3 | 1 |  |  |
| Not classified | -- | -- | 1 | 2 | 3  (1.1%) | -- | -- | -- | 1 | 3 | 4  (0.8%) | 7  (0.8%) |
| Total | -- | -- | 8 | 255 | 263 | 15 | 179 | 194 | 69 | 412 | 481 | 938 |

^1^ Colour was determined on dry artefacts in reference to Munsell Color System reference chips, published as the *Geological Society of America Rock Color Chart*.

**Table C. Cortex types on chert and chalcedony artefacts, all contexts, Horizon IV, Saruq al-Hadid.**

| **Colour group** | **Colour variety** | **Chert** | | | **Chalcedony** | | |
| --- | --- | --- | --- | --- | --- | --- | --- |
|  |  | **Flaw Line** ^1^ | **Hard** ^2^ | **Soft** ^3^ | **Flaw Line** ^1^ | **Hard** ^2^ | **Soft** ^3^ |
| Brown/Tan | Brown | 1 | 19 | 44 | 1 | -- | -- |
|  | Brown and red striped | -- | 4 | 4 | -- | -- | -- |
|  | Tan | 1 | 29 | 83 | 1 | -- | 1 |
|  | Tan and red striped | -- | 9 | 14 | -- | -- | -- |
| Total,  N (%) | -- | 2  (1.0%) | 61  (29.3%) | 145  (69.7%) | 2 | -- | 1 |
| Orange/Yellow | Orange | -- | 3 | -- | -- | -- | -- |
|  | Yellow | -- | 2 | 1 | -- | 3 | 4 |
| Total,  N (%) | -- | -- | 5  (83.3%) | 1  (16.7%) | -- | 3 | 4 |
| White/gray | White/gray | 5 | 18 | 12 | -- | 3 | 12 |
| Total,  N (%) | -- | 5  (14.3%) | 18  (51.4%) | 12  (34.3%) | -- | 3  (20.0%) | 12  (80.0%) |
| Red | Red | -- | 6 | 2 | -- | 2 | 1 |
| Total,  N (%) | -- | -- | 6  (75.0%) | 2  (25.0%) | -- | 2 | 1 |

^1^ ‘Flaw line’ cortex is a natural chemically-weathered surface that was exposed when a stone fractured along a joint. The surface is often relatively flat.

^2^ ‘Hard’ cortex is a mechanically or chemically weathered natural surface formed in colluvial deposits, and includes potlidding and patination. ‘Fluvial’ cortex, a type of hard cortex created by rolling in water, was noted on ophiolite and quartz artefacts, but was not observed on chert or chalcedony artefacts.

^3^ ‘Soft’ cortex is a chalky exterior marking the transition zone between the chert nodule and the parent limestone. The presence of flaw line and soft cortex suggests the stone was gathered at the bedrock exposure.

**Table D. Size range of crushing byproducts, metamorphic stone, contexts 1309 and 2008, Horizon IV, Saruq al-Hadid.**

| **Type** | **Dimensions (mm, Avg ± SD)** | | | |
| --- | --- | --- | --- | --- |
|  | **Length** | **Width** | **Thickness** | **Weight**  **(g)** |
| Crushed piece  (N=4) | 25.4 ± 11.8 | 23.2 ± 10.7 | 10.1 ± 6.3 | 10.4 ± 12.8 |
| Crushing flake  (N=7) | 7.4 ± 1.4 | 8.3 ± 3.2 | 3.1 ± 1.5 | 0.3 ± 0.3 |

**Table E. Number of platforms on cores and flake counts, contexts 1309 and 2008, Horizon IV, Saruq al-Hadid.**

| **Core type** | **Number of platforms** | | | **Total** |
| --- | --- | --- | --- | --- |
|  | **1**  **(not rotated)** | **2**  **(1 rotation)** | **3**  **(2 rotations)** |  |
| **Context 1309** | | | | |
| Single platform | 1 | -- | -- | 1 |
| Multiplatform | -- | 5 | 1 | 6 |
| Bifacial centripetal | 1 | -- | -- | 1 |
| Redirecting flakes | -- | -- | -- | 8 |
| Core reduction flakes | -- | -- | -- | 220 |
| **Context 2008** | | | | |
| Single platform | 1 | -- | -- | 1 |
| Multiplatform | -- | 1 | 1 | 2 |
| Bifacial centripetal | 2 | -- | -- | 2 |
| Redirecting flakes | -- | -- | -- | 10 |
| Core reduction flakes | -- | -- | -- | 170 |

**Table F. Colours of backed microliths compared to other artefact types, chert and chalcedony, Horizon IV, Saruq al-Hadid.**

| **Colour group**^1^ | **Reduction stage** | | **Other artefacts** |
| --- | --- | --- | --- |
|  | **Manufacturing rejects** | **Finished Microliths** |  |
| Brown/Tan | 101  (66.0%) | 72  (76.6%) | 486  (51.8%) |
| Orange/Yellow | 15  (9.8%) | 2  (2.1%) | 206  (22.0%) |
| White/Gray | 31  (20.3%) | 15  (16.0%) | 181  (19.3%) |
| Red | 5  (3.3%) | 0 | 45  (4.8%) |
| Other | 1  (0.7%) | 5  (5.3%) | 13  (1.4%) |
| Not classified | -- | -- | 7  (0.8%) |
| Total | 153  (100.1%) | 94  (100%) | 938  (100.1%) |

^1^ See S1 Tables, Table B for definitions of colour groups.
